# Presence 5 for trauma informed care: teaching tangible practices towards bidirectional healing in undergraduate medical education

**DOI:** 10.1186/s12909-025-08390-2

**Published:** 2025-12-05

**Authors:** Megan King, Zoe King, Donna Zulman, Megha Shankar

**Affiliations:** 1 University of California San Diego School of Medicine, San Diego, CA USA; 2 Harbor-UCLA Medical Center, Torrance, CA 90509 USA; 3 School of Population Health, University of New South Wales, Sydney, Australia; 4 Department of Medicine, Division of Primary Care and Population Health, Stanford University School of Medicine, Palo Alto, CA USA

**Keywords:** Trauma-informed care, Self-care, Medical education, Interpersonal and communication skills, Curriculum design and development

## Abstract

**Introduction:**

Trauma impacts health; the adverse health effects of trauma are well-understood and trauma-informed care is recommended to mitigate these effects. There is an opportunity to leverage evidence-based frameworks to add to the growing body of literature on teaching trauma-informed care within undergraduate medical education. To address this, we developed, implemented, and evaluated the Presence 5 for Trauma Informed Care Workshop (P5 TIC), a discussion-based workshop, informed by evidence and guided by a clinical case, to teach tangible trauma-informed care practices in undergraduate medical education.

**Methods:**

P5 TIC was developed by abstracting practices from a narrative literature review and mapping them to the Presence 5 framework. Evidence-based practices informed the development of a 1-hour, in-person workshop comprising didactics followed by a case-based discussion. P5 TIC was implemented with undergraduate medical students (*n* = 144) through the Practice of Medicine Course. Participants completed a pre- and post-workshop survey to evaluate confidence and attitudes related to trauma informed care.

**Results:**

Literature review resulted in the following P5 TIC practices: (1) Prepare with Intention (e.g., chart review mindfully) (2), Listen Intently and Completely (e.g., listen for signs and impacts of trauma when your patient is ready to share) (3) , Agree on What Matters Most (e.g., prioritize your patient’s trauma-related medical needs) (4), Connect with the Patient’s Story (e.g., reflect on how trauma intersects with healthcare) (5), Explore Emotional Cues (e.g., tune into body language and non-verbal cues), and (6)Care for Yourself (e.g., practice compassionate detachment). Learner confidence and attitudes related to trauma informed care increased after participation in P5 TIC.

**Conclusion:**

P5 TIC is a structured, evidence-based approach to teaching trauma informed care and adds to the literature in providing tangible practices as well as addressing self-care. Further medical education research should be conducted to explore the impact of P5 TIC on learner and patient outcomes.

**Supplementary Information:**

The online version contains supplementary material available at 10.1186/s12909-025-08390-2.

## Background

 Trauma results from an event, series of events, or set of circumstances that is experienced by an individual as physically or emotionally harmful with lasting adverse health effects [1]. There are a variety of trauma sources, such as adverse childhood experiences (ACEs), intimate partner violence (IPV), racism, and healthcare-induced trauma, among others. Adverse health effects of trauma include physical and mental health issues, as well as increased likelihood to experience other traumatic events, revictimization, increased ACEs score, and increased risk of developing depression or post-traumatic stress disorder (PTSD) [2]. Additionally, healthcare can be a place of re-traumatization, and those marginalized by the medical system may be reluctant to disclose histories of trauma due to an underlying distrust [2].

Trauma-informed care (TIC) is a practice that promotes the following six principles: [1] safety [2], trustworthiness and transparency [3], peer support [4], collaboration and mutuality [5], empowerment, voice, choice, and [6] cultural, historical, and gender issues [1]. Although these provide a theoretical framework for interacting with patients with a history of trauma, clinicians may benefit from tangible and practical approaches to trauma informed care in medical settings.

Teaching TIC within medical education is crucial to training future clinicians to provide effective, patient-centered care to diverse patient populations. There have been efforts to develop TIC curricula, though many are specific to a type of trauma (e.g., IPV) [3–5], portion of the clinical encounter (e.g., physical exam) [6], or medical specialty (e.g., Obstetrics and Gynecology) [7]. The literature is growing around generalized TIC education [6, 8–11], and there is an opportunity to bolster this by leveraging existing evidence-based frameworks to further promote a structured and practical approach to teaching TIC broadly within undergraduate medical education.

Frameworks that focus on humanism may provide an effective scaffolding to teach TIC broadly in undergraduate medical education. Presence 5 (P5) is an evidence-based framework that promotes humanistic communication between clinicians and patients through tangible practices to utilize in interpersonal interactions [12]. The five P5 practices are: Prepare with Intention, Listen Intently and Completely, Connect with the Patient’s Story, Agree on What Matters Most, and Explore Emotional Cues. The P5 framework has been successfully adapted to multiple medical and educational settings including telemedicine, racial justice, and pediatrics [13–18]. As such, P5 may be an effective framework to adapt and teach TIC in medical education.

To advance efforts in teaching tangible, evidence-based, and generalized TIC practices in medical education, the objectives of this study were to [1] adapt P5 to TIC as an educational innovation, developing the Presence 5 for Trauma Informed Care Workshop (P5 TIC), and [2] pilot P5 TIC within undergraduate medical education, evaluating learner confidence and attitudes in TIC.

## Methods

This pilot medical education innovation involves the development, implementation, and evaluation of P5 TIC during the 2022–2023 academic year at a single urban, academic institution. Participation was voluntary and written informed consent was obtained from all participants. There was no incentive for survey completion. The study was deemed Exempt by the University of California San Diego IRB #802,618.

### P5 TIC development

To develop P5 TIC, we first conducted a narrative literature review using the search terms “trauma-informed care” AND “ACEs” OR “gender-based violence,” OR “intimate partner violence,” OR “LGBTQIA,” OR “HIV,” OR “sex trafficking,” OR “refugee health,” OR “incarceration” OR “racism” OR “healthcare-induced trauma.” Inclusion criteria were papers published in English, up to the year 2023, systematic reviews, or primary literature on tangible trauma-informed care practices. Common themes from TIC practices were identified and mapped to one or more of the five practices of the P5 framework [2, 19–31]. Literature was reviewed by 3 authors, MK, ZK, and MS.

A 35-slide presentation (Supplement 1) was developed from the literature review findings for content and based off a previous P5 workshop for structure (18). The 1-hour workshop included a 10-minute introductory didactic section reviewing the evidence-based practices, 5-minute self-reflection exercise, 40-minute case-based discussion, and 5-minute debrief with goal setting.

During the initial P5 TIC workshop as part of a pre-clinical medical elective on TIC, learners highlighted the need for self-care strategies as an important aspect of practicing TIC in clinical settings; thus, we iteratively added to the narrative literature review and included strategies for self-care and preventing vicarious trauma. The literature review included search terms “self-care” AND “vicarious trauma.” Common practices were then selected to synthesize evidence-based practices for implementing self-care strategies when caring for patient who have experienced trauma [32–37]. A self-care component was added to the adapted P5 TIC framework.

### P5 TIC implementation

P5 TIC was implemented in undergraduate medical education at a single urban, academic institution. An initial workshop was given to students enrolled in a School of Medicine elective course entitled “Intersectional Approaches to Trauma-Informed Care.” As described above, the workshop content was refined based on initial feedback and a revised P5 TIC was implemented to all first-year medical students through the Practice of Medicine Course.

### P5 TIC evaluation

Pre- and post-workshop online surveys (Supplement 2) were administered to measure confidence and attitudes towards TIC before and after participation in P5 TIC. Participant demographics were collected in the pre-workshop survey. TIC confidence was evaluated with participant self-reported confidence in three author-developed learning objectives using a 4-point Likert-scale for participants to choose either a positive or negative response [38]. The validated ARTIC-10 scale was used [39] to evaluate attitudes related to trauma-informed care. The ARTIC-10 scale is a brief, 10-item tool designed to measure individuals’ attitudes related to TIC across human service, education, and healthcare settings, providing an overall score reflecting acceptance and support of TIC principles. We used this scale to assess learner perception towards TIC in medical education and followed the recommended validated language of the scale accordingly. Pre- and post-workshop survey data were analyzed using summary statistics and the Welch Two Sample t-test. Finally, open-ended feedback on P5 TIC was collected in the post-workshop survey.

## Results

### Literature review results

**Fig. 1:**
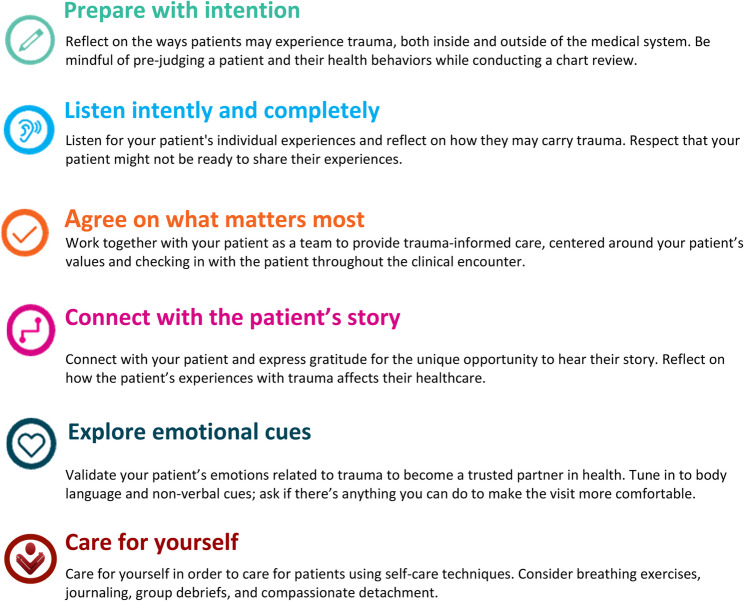
Overview of evidence-based practices for promoting trauma informed care in clinical encounters, adapted from the original Presence 5 framework

Through narrative literature review, P5 TIC practices were identified; Fig. 1, entitled “Presence 5 for Trauma-Informed Care,” displays an overview of evidence-based practices for promoting trauma-informed care in clinical encounters, adapted with permission from the original P5 framework [12]. Original P5 practices include: [1] Prepare with Intention [2], Listen Intently and Completely [3], Agree on What Matters Most [4], Connect with the Patient’s Story, and [5] Explore Emotional Cures. When adapted to TIC, our findings included several practices for each of the P5, as well as an additional practice on self-care: [6] Care for Yourself. To Prepare with Intention, results showed that clinicians should be mindful of word choice and use non- judgemental, neutral, and patient-centred language [2, 19, 29, 30]. This practice also includes familiarizing oneself with clinical or institutional guidelines about screening for trauma and follow-up resources (i.e. social work, safety planning, legal involvement). To Listen Intently and Completely, the evidence suggests that using active listening can counteract feelings of coercion that is often experienced in gender-based trauma. Allow patients to tell stories in the own way, if and when they are ready. This often requires the use of open-ended questions to probe health related information (i.e. “Has anything in your life affected your health and well-being?”). However, providers should be cautious of exploring a patient’s psychological history if they do not have trauma-specific training [2, 19, 20, 29]. To Agree on What Matters Most, efforts to empower the patient’s goals, values, and strengths should occur early on and periodically throughout the clinical encounter, as patients with histories of trauma report a wide variety of concerns in a healthcare setting [2]. Prior to physical exams, outline the steps of the exam and inform the patient on what to expect, and remind the patient they can say “no” or “stop” anytime. To Connect with the Patient’s Story, clinicians should focus on taking a multidisciplinary and network-oriented approach to patient care to re-emphasize the importance of community in healing from traumatic events [2, 19, 27, 29, 31]. Reframe maladaptive coping behaviors as responses to trauma, and suggest alternative coping strategies (i.e. journaling, exercise, social support). To Explore Emotional Cues, pay attention to both verbal and non-verbal communication of the patient and reflecting, validating, and confirming perceptions of the patient’s emotions (i.e. “I can see this is affecting you deeply”) helps patients understand their response to specific trauma and validation can serve as a powerful coping tool. Importantly, physicians and other healthcare providers are at increased of developing vicarious trauma [32, 33]. To Care for Yourself, the literature emphasized the need for practice compassionate detachment, a practice that maintains empathy and compassion while establishing emotional boundaries to protect one’s own emotional and mental well-being. Other practices include participating in group debriefs, using cognitive-behavioural exercises or grounding techniques, and creative writing [40].

### Survey results

144 pre-clinical medical students participated in P5 TIC with pre- and post-workshop survey response rates of 27.8% (*N* = 40) and 17.4% (*N* = 25), respectively. Participants identified as ages 18–30 [52.5%], women [65.0%], and white [32.5%] (Table 1). Table 2 shows the pre- and post-workshop survey responses regarding TIC confidence and attitudes. Participation in P5 TIC led to a statistically significant increase in participant confidence across all three learning objectives (*p* < 0.005). There was a statistically insignificant increase in attitudes related to trauma informed care by the ARTIC scale (*p* >0.05) [39], where higher mean ARTIC scores signify a more positive attitude towards practicing trauma informed care. Open-ended feedback showed a generally positive experience with P5 TIC.

**Table 1:** Demographics of P5 TIC Survey Respondents

Demographic Category	Number* (*N* = 40)	Percentage (%)
Age		
18 - 30	21	52.5
31 - 40	5	12.5
Gender		
Woman	26	65.0
Man	3	7.5
Race/Ethnicity		
White	13	32.5
Asian	9	22.5
Middle Eastern/North African; Hispanic, Latino, or Spanish Origin; Black or African American**	4	10.0

Demographics of learners who participated in P5 TIC and completed the pre-workshop survey

*Number of participants in each demographic category may not add up to 40 as participants were not required to answer all demographic questions

** Participants who identified as Middle Eastern/North African; Hispanic, Latino, or Spanish Origin; Black or African American were combined into one demographic category to preserve anonymity as some of these categories had one respondent

**Table 2:** Learner Confidence and Attitudes Related to Trauma-Informed Care Before and After Participation in P5 TIC

	Pre-Workshop (*N* = 40)	Post-Workshop (*N* = 25)	*p*-value	95% Confidence Interval
TIC Confidence				
How confident are you in your ability to define trauma and name different types of traumatic experiences?	2.46	3.00	0.00021	[−0.867, −0.283]
How confident are you in your ability to understand how to use evidence-based practices for trauma-informed care in a clinical setting?	1.95	2.81	0.00002	[−1.206,−0.494]
How confident are you in your ability to respond to patients when they disclose a history of trauma in a way that is empowering and avoids re-traumatization?	2.16	2.77	0.00079	[−0.876, −0.244]
TIC Attitudes				
ARTIC-10 Mean Score	5.77	6.04	0.095	[−0.579, 0.047]

Learner confidence and attitudes related to trauma informed care before and after participation in P5 TIC. The survey questions ask about learner confidence in the three learning objectives, using a 4-point Likert scale, where 1 = not confident at all, 2 = not very confident, 3 = fairly confident, 4 = very confident. Attitudes related to trauma-informed care (ARTIC) were measured using the ARTIC-10 scale. ARTIC-10 is a 10-item short form for a measure of attitudes toward trauma-informed care that uses a bipolar Likert scale, where items are scored from 1 to 7. Mean scores are used, and higher scores demonstrate more favorable attitudes towards trauma-informed care. *P*-values were calculated using the Welch Two Sample t-test, where statistical significance was defined at *p*<0.05.

## Discussion

We adapted the P5 framework to TIC, developing an educational innovation, P5 TIC, that was implemented as a pilot in a single urban, academic institution. P5 TIC can be utilized to teach trauma-informed clinical skills and trauma-informed self-care strategies within undergraduate medical education, with pilot data suggesting positive effects on learner confidence level and attitudes towards TIC.

In this pilot innovation, P5 TIC shows promise of being an effective and feasible educational intervention. Learner self-reported confidence in all three TIC learning objectives increased significantly, demonstrating a preliminary effectiveness in P5 TIC as a knowledge-enhancing intervention. As such, this pilot innovation adds to the growing literature in TIC curricula across the country, adding a structured and evidence-based framework that can be replicated by other institutions. Further, P5 TIC appears to be feasible within the context of undergraduate medical education. As a one-hour, interactive workshop that was integrated into existing curricular structures, P5 TIC can be implemented in a practical and sustainable manner that does not require major curricular re-structuring. Evidence shows that new educational interventions that are discussion-based, time-limited, and integrated into existing curricular structures are important factors for them to be feasible and sustainable [41]. Additionally, P5 TIC was co-created with learners and faculty, increasing the likelihood of developing an effective, feasible, and sustainable educational innovation. Evidence in the literature supports the approach of co-creating new curricula with learners to empower and engage learners, promote academic careers, and enhance learner wellbeing [42, 43].

Curricula evaluation is a crucial component of furthering medical education efforts. We used ARTIC, a validated and reliable measurement for determining attitudes related to trauma-informed care in healthcare providers [39]. It has been utilized in various settings including social services, schools, and substance-use disorder treatment [44]. Although ARTIC is widely used to assess attitudes related to TIC, there lacks sufficient research demonstrating a quantifiable increase in patient outcomes or satisfaction.

An advantage of this study was its ability to mesh the original P5 framework with principals of TIC to create tangible practices for learners, building a foundation for scaling up. Existing TIC frameworks align with P5 TIC produced through the narrative review. For example, the Substance Abuse and Mental Health Services Administration (SAMHSA) developed their six guiding principles to TIC and Four “R”s (realize, recognize, respond, and resist), and the recommended clinical practices in P5 TIC align across these frameworks [1]. For example, taking a “network-oriented” approach that recognizes the patient’s community as an integral part of the healing process aligns with the principle of peer support. Overlap between existing TIC frameworks and P5 TIC help to validate its usefulness while adding to these frameworks by providing actionable steps that learners can take to implement TIC.

The P5 TIC innovation highlights the importance of the additional, sixth element of P5 TIC, self-care, which is critical to medical education around TIC. Caring for patients with trauma may result in learners experiencing vicarious trauma and can lead to burnout [33]. The original Presence 5 framework acknowledged how improving clinician communication strategies led to improved self-care for clinicians [12, 16]. The P5 TIC adaptation provides a unique opportunity to further address the need to support both patients and learners in the clinical encounter due to risk of re-traumatization of patients and vicarious trauma for learners. With trauma-informed self-care as a sixth practice, some of the P5 TIC clinical practices that address vicarious trauma may depart from the original P5 framework. For example, in the Listen Intently and Completely domain, compassionate detachment is a P5 TIC self-care strategy in which the clinician may choose not to physically lean in while listening to the patient’s story, in effort to protect themselves from vicarious trauma [40]. While this P5 TIC recommendation differs from the original P5 recommendation of physically leaning in as a communication skill [12], it may be a necessary self-care strategy to prevent vicarious trauma [34–37]. A review on vicarious trauma interventions for service providers presents additional interventions that generally reduced secondary trauma stress and burnout, such as psychoeducation and mindfulness interventions [40]. Building on the original P5 framework, P5 TIC highlights the additional importance of supporting learners from an institutional level, as well as individual-level interventions, in self-care practices to ensure learners are adequately supported to treat patients who have experienced trauma.

While the P5 TIC curriculum demonstrates a promising step in the direction of actionable TIC practices, it is not without limitations. First, P5 TIC practices have not yet been evaluated with respect to patient outcomes. Second, the sample size was small as participants were restricted to enrolled medical students from one academic institution and the low response rate of the survey may affect the significance of the results. Additionally, due to unmatched participant responses in the pre- and post- surveys, data are limited by the inability to match pairs and directly measure the change in attitudes and knowledge for individual participants. Finally, we received open-ended feedback from learners but did not perform formal qualitative analysis due to limited number of comments. Future implementation practices could consider collecting more robust qualitative data with subsequent analysis to further explore learner experiences with P5 TIC and impact on medical education.

## Conclusions

P5 TIC is a pilot educational innovation that adds to the growing body of literature of practical, structured tools for learners to support patients who have experienced trauma. The successful adaptation of the P5 framework to TIC and the increase in self-reported confidence in TIC skills among learners underscores the potential for P5 TIC to be scaled within medical education. Further research is needed to study the implementation of P5 TIC in other medical education settings, as well as the impact of P5 TIC on health outcomes for patients who have experienced trauma.

## Supplementary Information

Supplementary Material 1.

Supplementary Material 2.

## Data Availability

The datasets used and/or analyzed during the current study are available from the corresponding author on reasonable request.
