# Supplementary material for: Presence 5 for trauma informed care: teaching tangible practices towards bidirectional healing in undergraduate medical education
Source: BMC Med Educ. 2025 Dec 5;26:45. doi: 10.1186/s12909-025-08390-2 (PMC12798035; doi:10.1186/s12909-025-08390-2)
Supplement: Supplementary file 1 — Supplementary Material 1. [file 12909_2025_8390_MOESM1_ESM.docx]

**Appendix B: Trauma-Informed Care Pre-Survey**

I. Trauma Informed Care Survey Consent

We are interested in assessing knowledge and attitudes related to trauma informed care. This survey will take about 5 minutes to complete. Your participation is completely voluntary, and you can stop at any time. Your responses are confidential, and we will not be using any personal identifiers that would link you to your responses. Please answer these questions as honestly as possible as your responses will help guide future trauma informed care education. Do you consent to this survey?

- Yes
- No

II. Confidence in Trauma-Informed Care

How confident are you in your ability to:

|  | not confident at all | not very confident | fairly confident | very confident |
| --- | --- | --- | --- | --- |
| Define trauma and name different types of traumatic experiences. |  |  |  |  |
| Understand how to use evidence-based practices for trauma-informed care in a clinical setting. |  |  |  |  |
| Respond to patients when they disclose a history of trauma in a way that is empowering and avoids re-traumatization. |  |  |  |  |

III. Attitudes Related to Trauma Informed Care

Author-adapted version of ARTIC-10 scale: Baker, C. N., Brown, S. M., Overstreet, S., Wilcox, P. D., & New Orleans Trauma-Informed Schools Learning Collaborative. (2021). Validation of the Attitudes Related to Trauma-Informed Care Scale (ARTIC). Psychological Trauma: Theory, Research, Practice and Policy, 13(5), 505–513. <https://doi.org/10.1037/tra0000989>

IV. Demographics

What is your current role?

- Medical Student
- Resident
- Prefer not to answer

What is your race/ethnicity? Please select all that apply.

- American Indian or Alaska Native (A person having origins in any of the original peoples of North and South America including Central America, and who maintains tribal affiliation or community attachment)
- Asian (A person having origins in any of the original peoples of the Far East, Southeast Asia, or the Indian subcontinent including, for example, Cambodia, China, India, Japan, Korea, Malaysia, Pakistan, the Philippine Islands, Thailand, and Vietnam)
- Black or African American (A person having origins in any of the black racial groups of Africa)
- Hispanic or Latino (A person of Cuban, Mexican, Puerto Rican, South or Central American, or other Spanish culture or origin)
- Middle Eastern or North African (A person having origins in any of the original people of the Middle East or North African, for example, Lebanon and Egypt)

Native Hawaiian or Other Pacific Islander (A person having origins in any of the original peoples of Hawaii, Guam, Samoa, or other Pacific Islands)

- Write-In __________________________________________________
- Prefer not to answer

What is your gender?

- Man
- Woman
- Non-binary/third gender
- Write-In __________________________________________________
- Prefer not to answer

What is your age?

- 18-30
- 31-40
- 41+
- Prefer not to answer

**Appendix B: Trauma-Informed Care Post-Survey**

I. Trauma Informed Care Survey Consent

We are interested in assessing knowledge and attitudes related to trauma informed care. This survey will take about 5 minutes to complete. Your participation is completely voluntary, and you can stop at any time. Your responses are confidential, and we will not be using any personal identifiers that would link you to your responses. Please answer these questions as honestly as possible as your responses will help guide future trauma informed care education. Do you consent to this survey?

- Yes
- No

II. Confidence in Trauma-Informed Care

How confident are you in your ability to:

|  | not confident at all | not very confident | fairly confident | very confident |
| --- | --- | --- | --- | --- |
| Define trauma and name different types of traumatic experiences. |  |  |  |  |
| Understand how to use evidence-based practices for trauma-informed care in a clinical setting. |  |  |  |  |
| Respond to patients when they disclose a history of trauma in a way that is empowering and avoids re-traumatization. |  |  |  |  |

III. Attitudes Related to Trauma Informed Care

Author-adapted version of ARTIC-10 scale: Baker, C. N., Brown, S. M., Overstreet, S., Wilcox, P. D., & New Orleans Trauma-Informed Schools Learning Collaborative. (2021). Validation of the Attitudes Related to Trauma-Informed Care Scale (ARTIC). Psychological Trauma: Theory, Research, Practice and Policy, 13(5), 505–513. <https://doi.org/10.1037/tra0000989>

IV. Feedback

Please feel free to share any comments, reflections, or questions on the Presence 5 for Trauma Informed Care Curriculum in the space below.
