# Supplementary material for: Presence 5 for trauma informed care: teaching tangible practices towards bidirectional healing in undergraduate medical education
Source: BMC Med Educ. 2025 Dec 5;26:45. doi: 10.1186/s12909-025-08390-2 (PMC12798035; doi:10.1186/s12909-025-08390-2)
Supplement: Supplementary file 2 — Supplementary Material 2. [file 12909_2025_8390_MOESM2_ESM.pptx]

## Slide 1
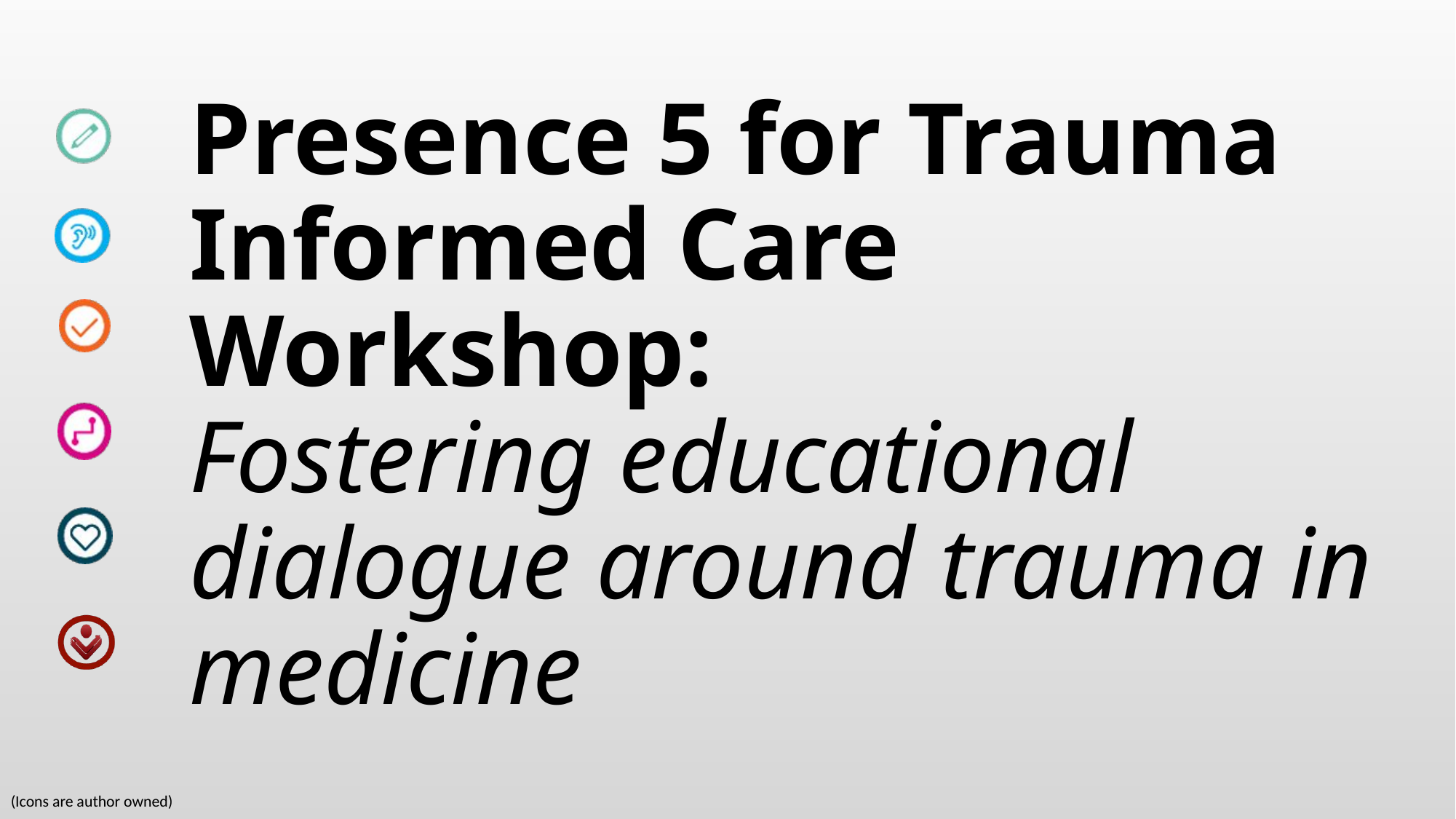

# Presence 5 for Trauma Informed Care Workshop: Fostering educational dialogue around trauma in medicine
(Icons are author owned)

## Slide 2
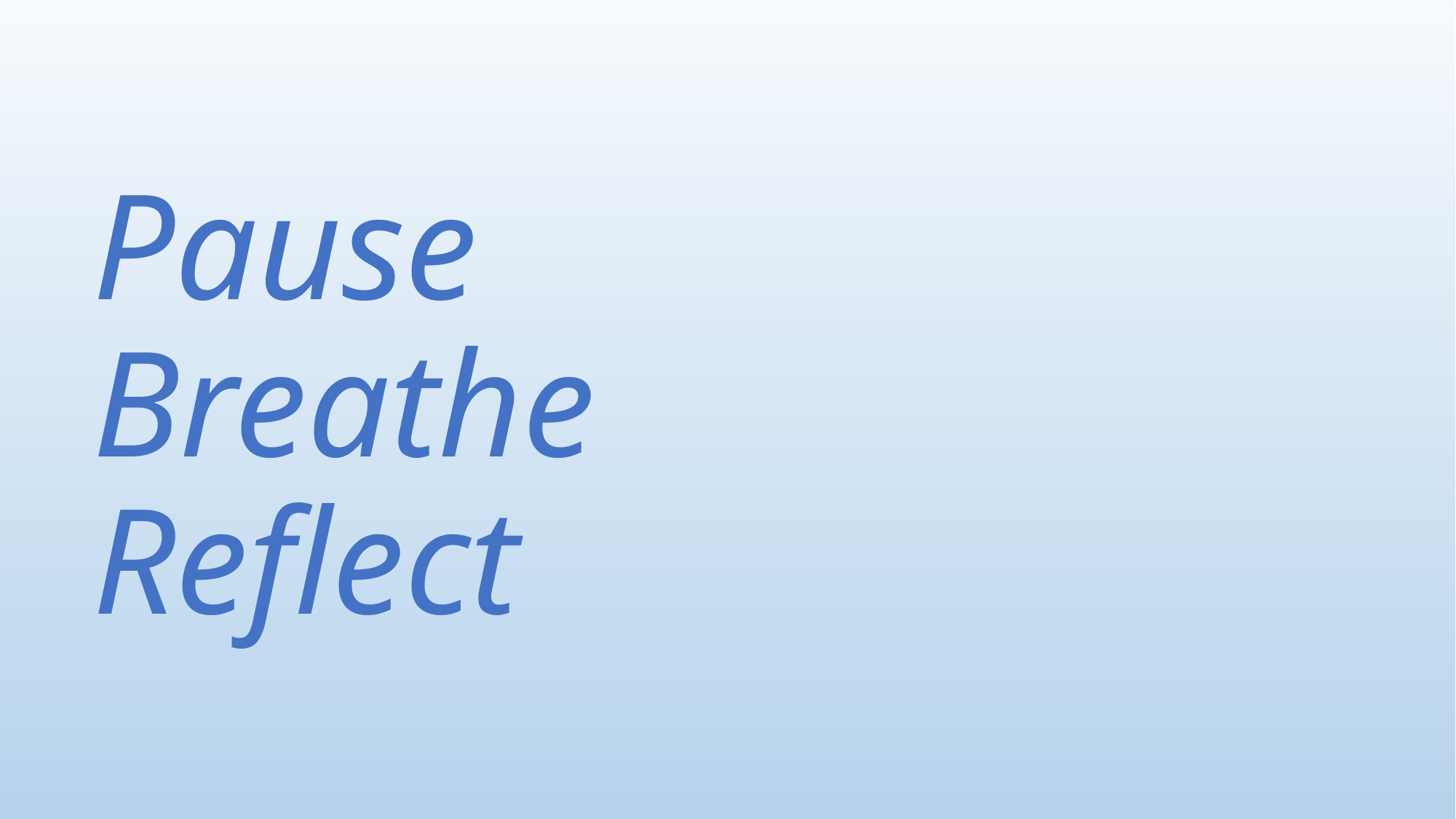

# Pause Breathe Reflect

## Slide 3
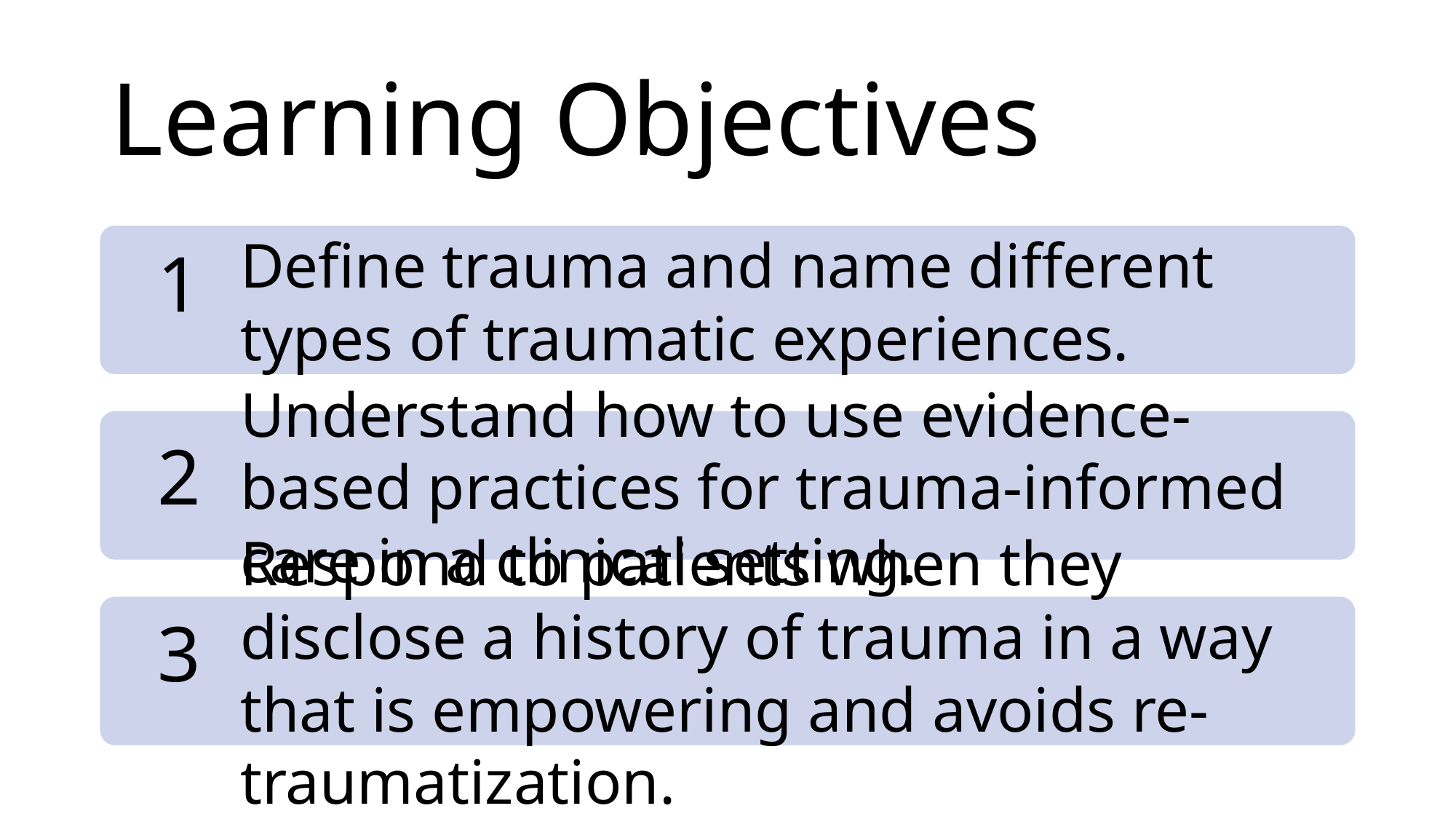

# Learning Objectives
Define trauma and name different types of traumatic experiences.
Understand how to use evidence-based practices for trauma-informed care in a clinical setting.
Respond to patients when they disclose a history of trauma in a way that is empowering and avoids re-traumatization.
1
2
3

## Slide 4
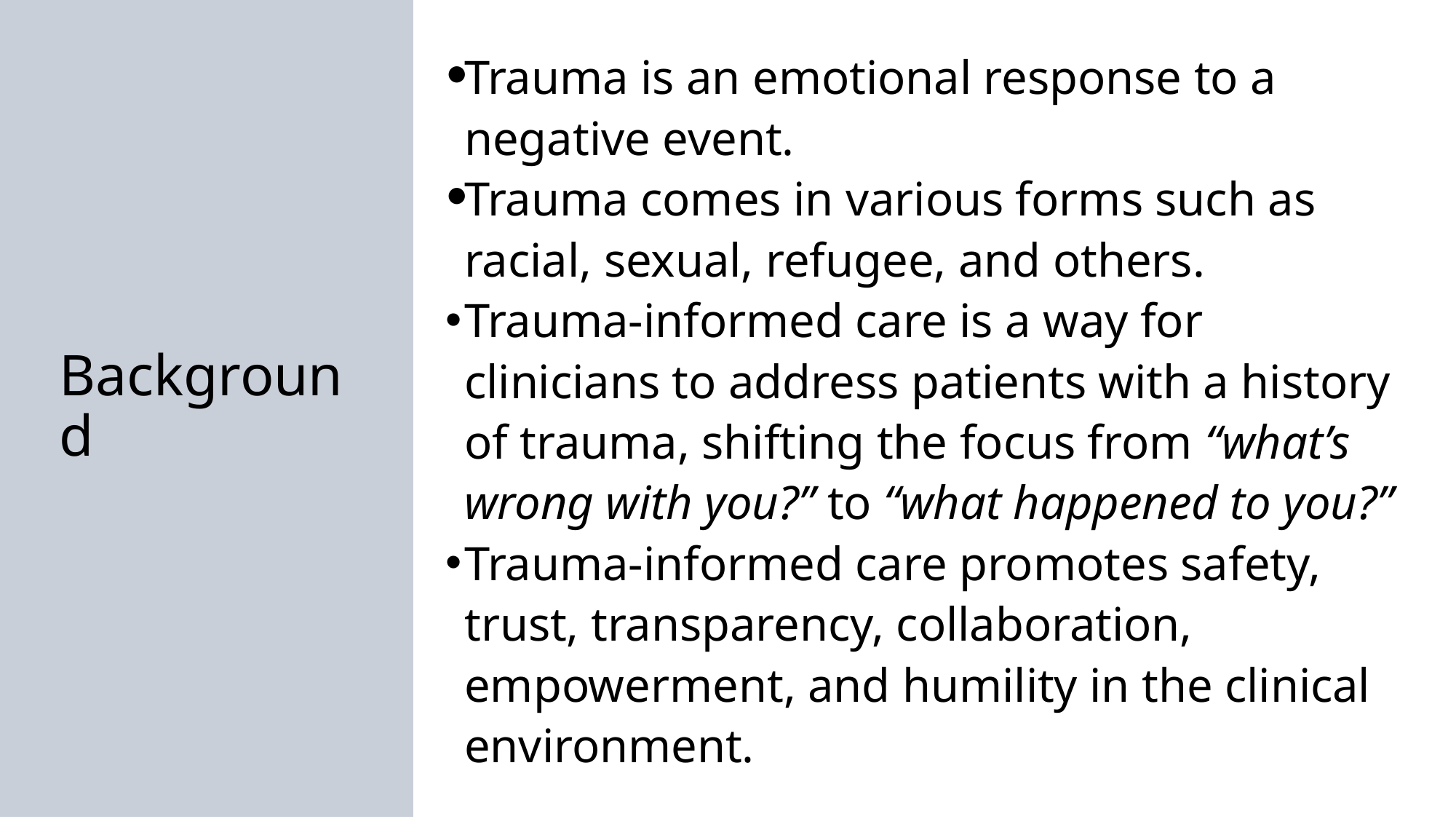

Trauma is an emotional response to a negative event.
Trauma comes in various forms such as racial, sexual, refugee, and others.
Trauma-informed care is a way for clinicians to address patients with a history of trauma, shifting the focus from “what’s wrong with you?” to “what happened to you?”
Trauma-informed care promotes safety, trust, transparency, collaboration, empowerment, and humility in the clinical environment.
# Background

## Slide 5
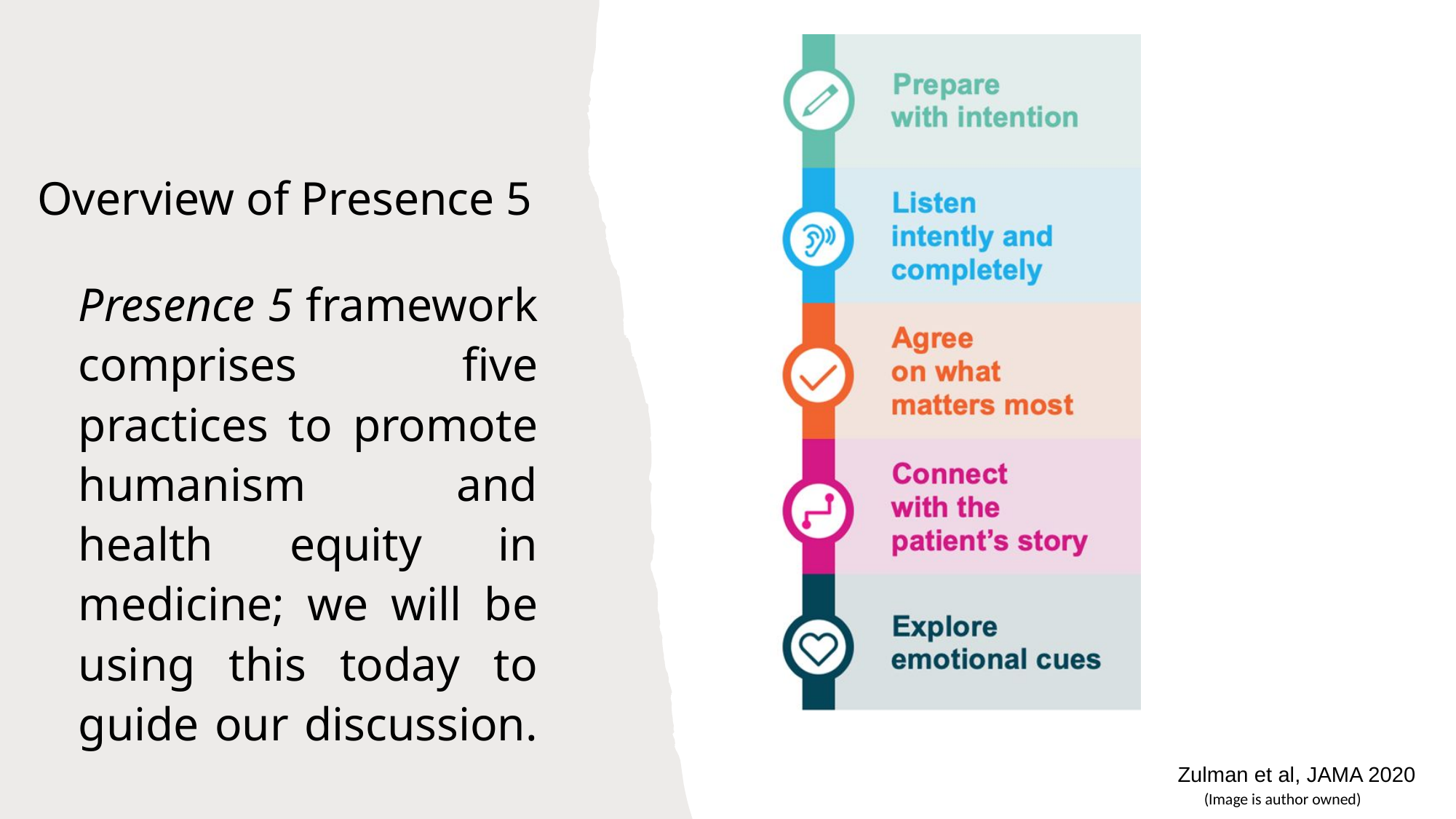

# Overview of Presence 5
Presence 5 framework comprises five practices to promote humanism and health equity in medicine; we will be using this today to guide our discussion.
Zulman et al, JAMA 2020
(Image is author owned)

## Slide 6
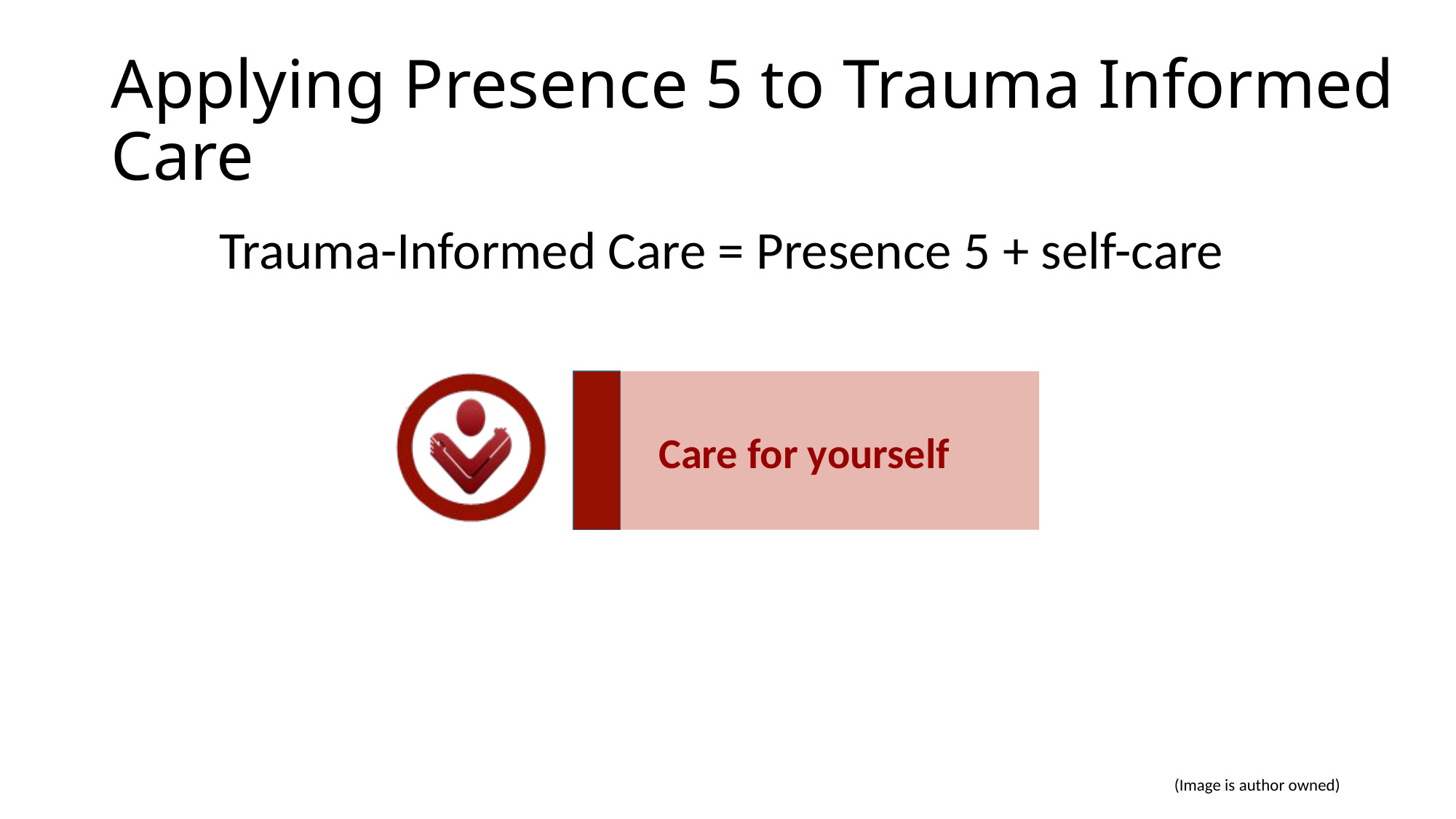

# Applying Presence 5 to Trauma Informed Care
Trauma-Informed Care = Presence 5 + self-care
Care for yourself
(Image is author owned)

## Slide 7
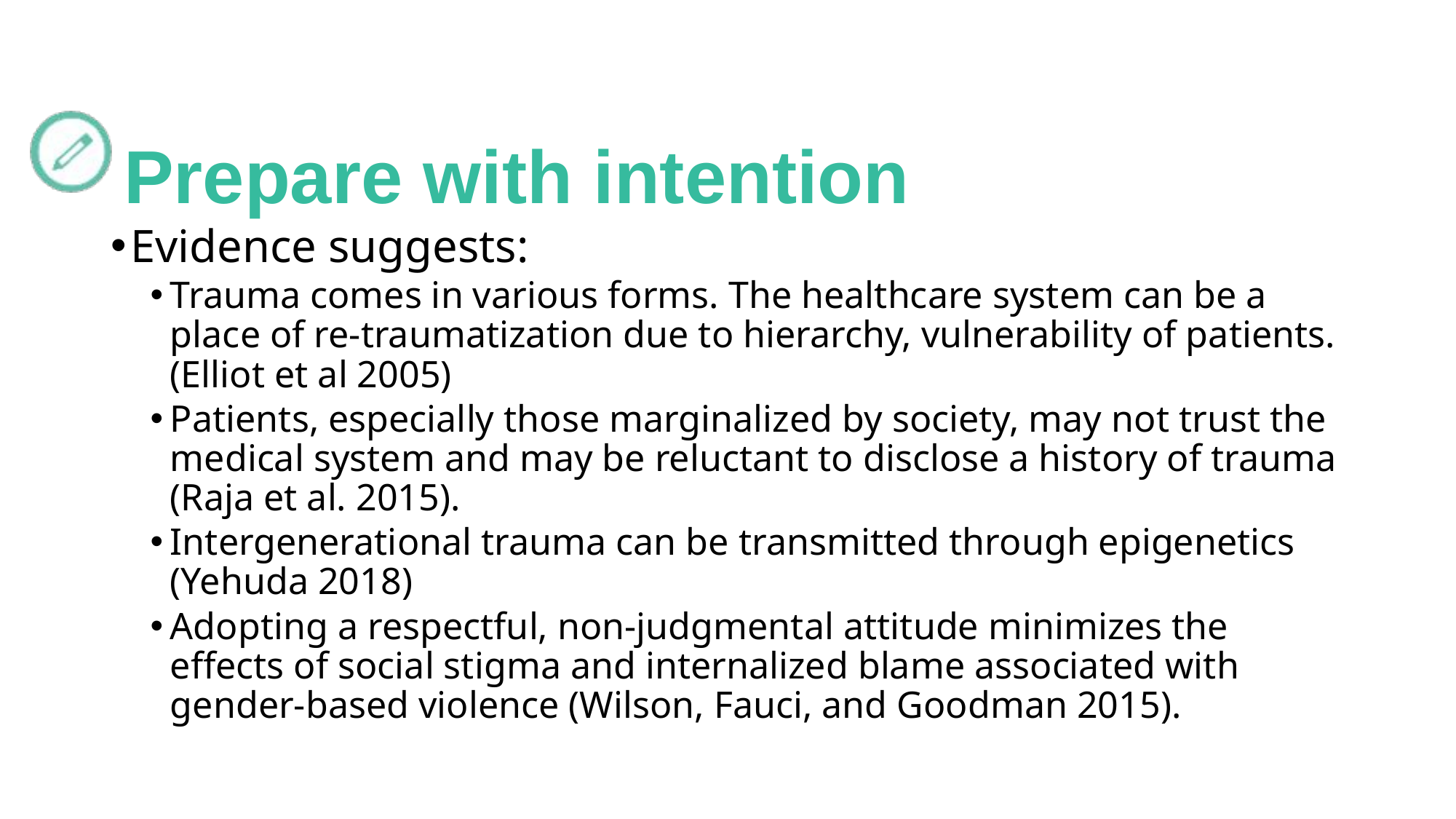

# Prepare with intention
Evidence suggests:
Trauma comes in various forms. The healthcare system can be a place of re-traumatization due to hierarchy, vulnerability of patients. (Elliot et al 2005)
Patients, especially those marginalized by society, may not trust the medical system and may be reluctant to disclose a history of trauma (Raja et al. 2015).
Intergenerational trauma can be transmitted through epigenetics (Yehuda 2018)
Adopting a respectful, non-judgmental attitude minimizes the effects of social stigma and internalized blame associated with gender-based violence (Wilson, Fauci, and Goodman 2015).

## Slide 8
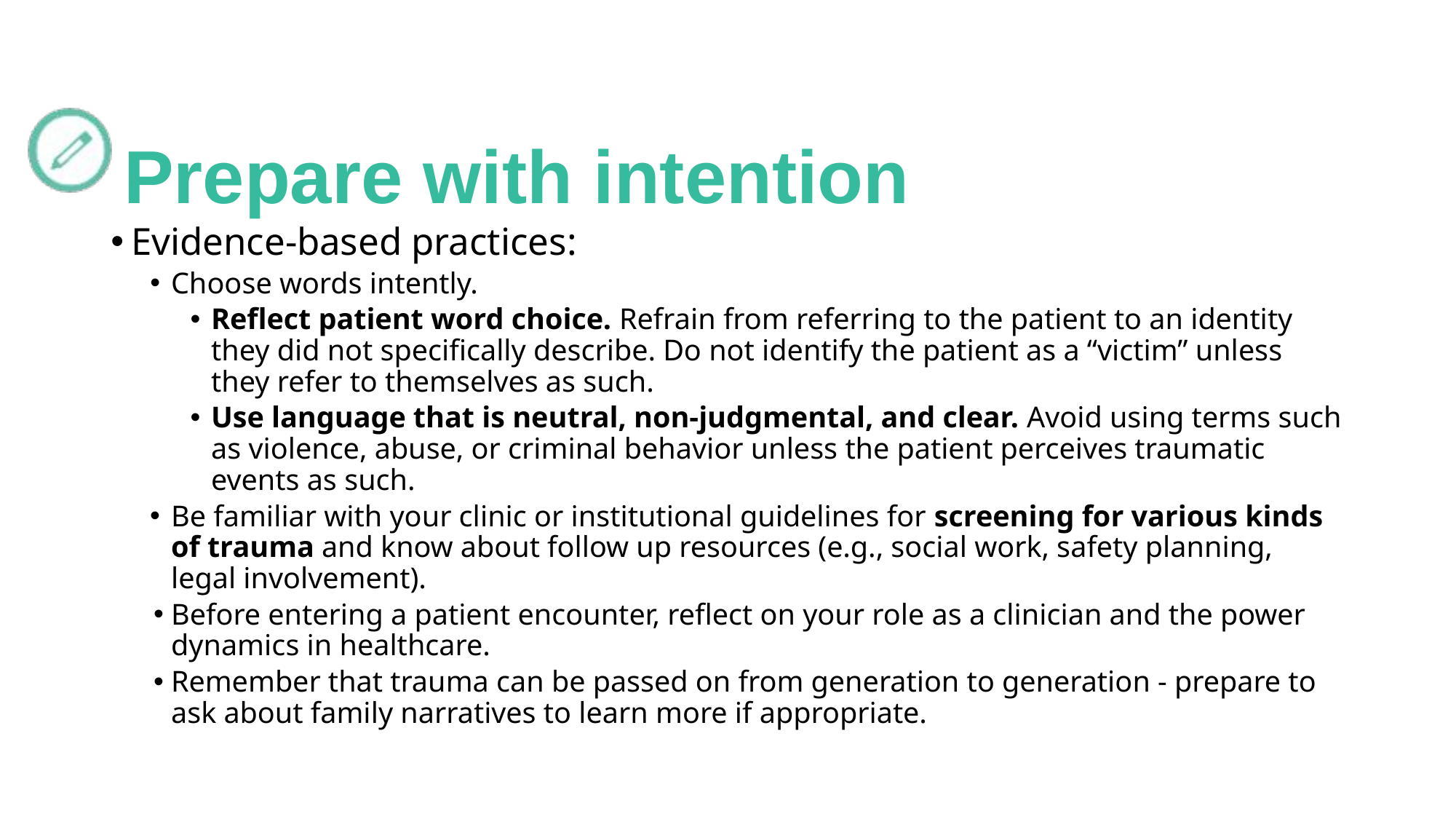

# Prepare with intention
Evidence-based practices:
Choose words intently.
Reflect patient word choice. Refrain from referring to the patient to an identity they did not specifically describe. Do not identify the patient as a “victim” unless they refer to themselves as such.
Use language that is neutral, non-judgmental, and clear. Avoid using terms such as violence, abuse, or criminal behavior unless the patient perceives traumatic events as such.
Be familiar with your clinic or institutional guidelines for screening for various kinds of trauma and know about follow up resources (e.g., social work, safety planning, legal involvement).
Before entering a patient encounter, reflect on your role as a clinician and the power dynamics in healthcare.
Remember that trauma can be passed on from generation to generation - prepare to ask about family narratives to learn more if appropriate.

## Slide 9
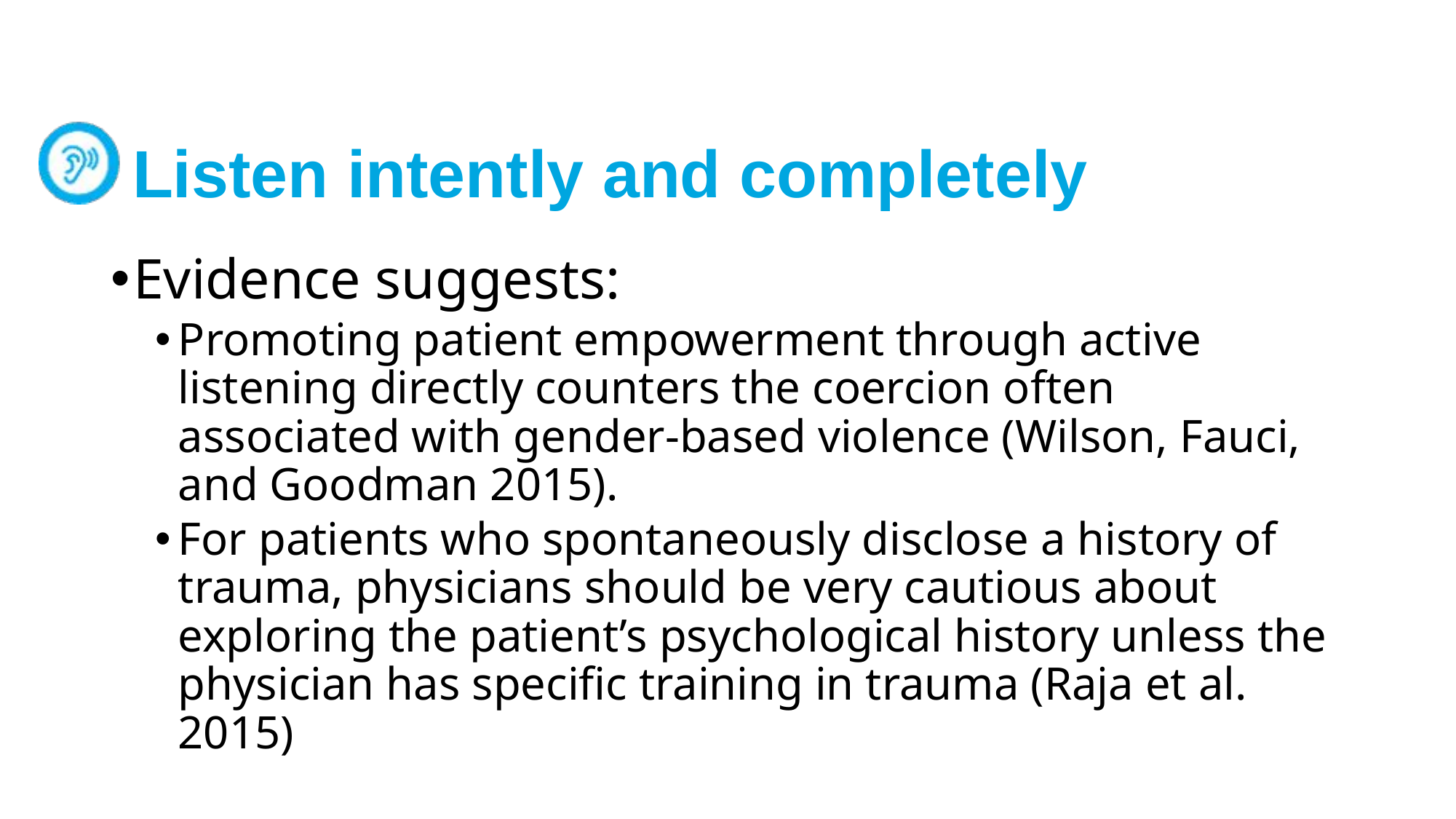

# Listen intently and completely
Evidence suggests:
Promoting patient empowerment through active listening directly counters the coercion often associated with gender-based violence (Wilson, Fauci, and Goodman 2015).
For patients who spontaneously disclose a history of trauma, physicians should be very cautious about exploring the patient’s psychological history unless the physician has specific training in trauma (Raja et al. 2015)

## Slide 10
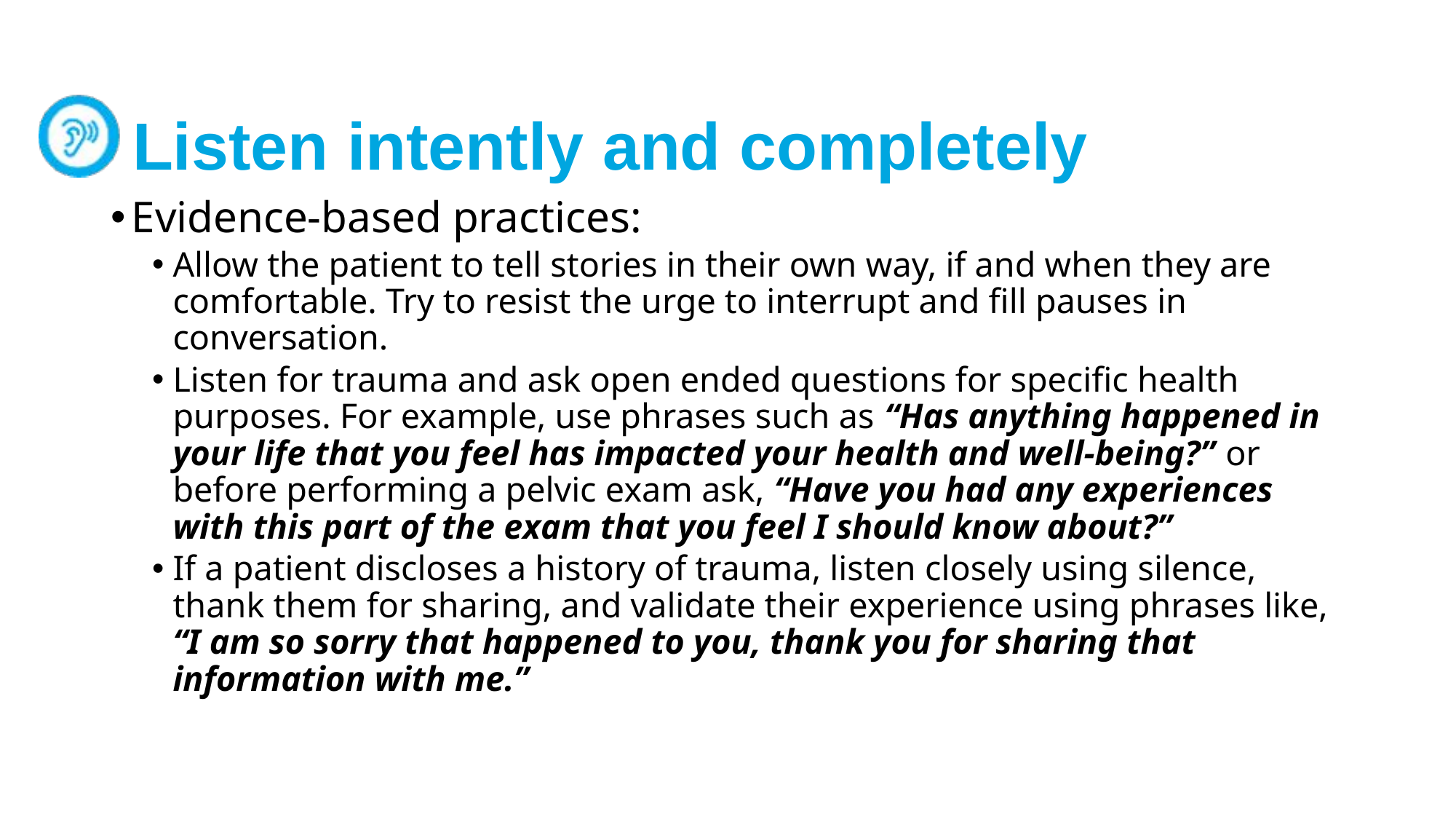

# Listen intently and completely
Evidence-based practices:
Allow the patient to tell stories in their own way, if and when they are comfortable. Try to resist the urge to interrupt and fill pauses in conversation.
Listen for trauma and ask open ended questions for specific health purposes. For example, use phrases such as “Has anything happened in your life that you feel has impacted your health and well-being?” or before performing a pelvic exam ask, “Have you had any experiences with this part of the exam that you feel I should know about?”
If a patient discloses a history of trauma, listen closely using silence, thank them for sharing, and validate their experience using phrases like, “I am so sorry that happened to you, thank you for sharing that information with me.”

## Slide 11
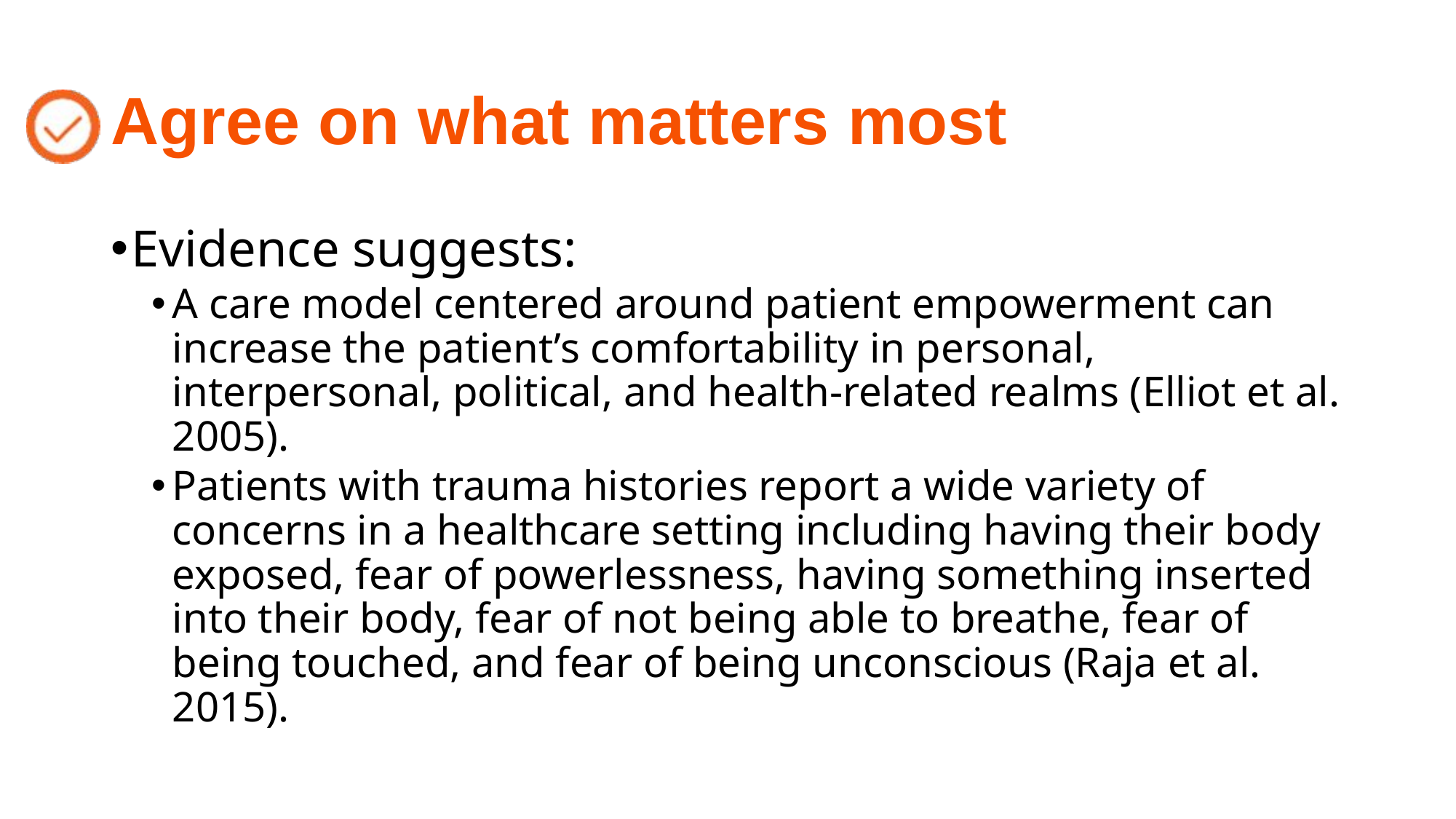

# Agree on what matters most
Evidence suggests:
A care model centered around patient empowerment can increase the patient’s comfortability in personal, interpersonal, political, and health-related realms (Elliot et al. 2005).
Patients with trauma histories report a wide variety of concerns in a healthcare setting including having their body exposed, fear of powerlessness, having something inserted into their body, fear of not being able to breathe, fear of being touched, and fear of being unconscious (Raja et al. 2015).

## Slide 12
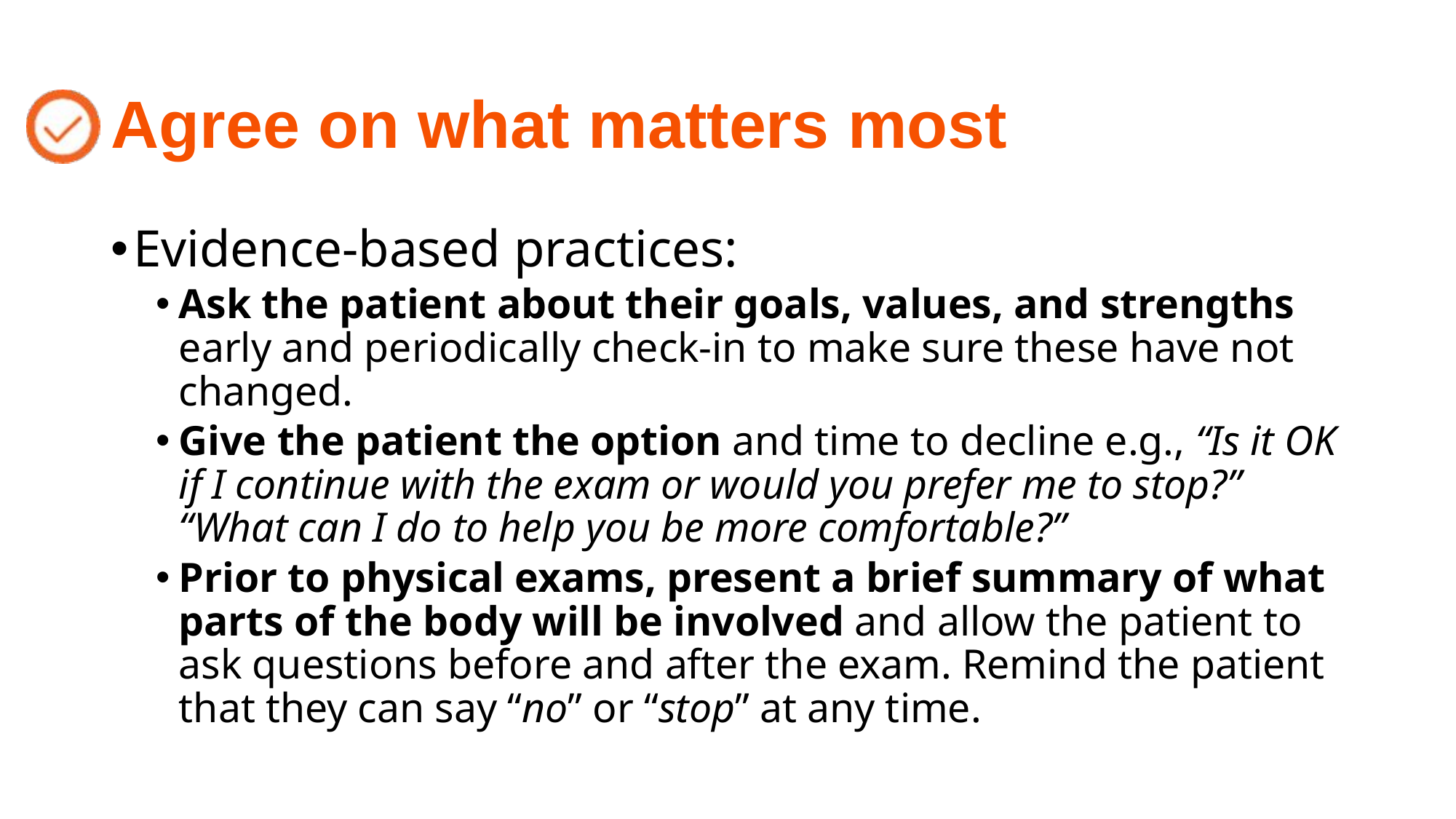

# Agree on what matters most
Evidence-based practices:
Ask the patient about their goals, values, and strengths early and periodically check-in to make sure these have not changed.
Give the patient the option and time to decline e.g., “Is it OK if I continue with the exam or would you prefer me to stop?” “What can I do to help you be more comfortable?”
Prior to physical exams, present a brief summary of what parts of the body will be involved and allow the patient to ask questions before and after the exam. Remind the patient that they can say “no” or “stop” at any time.

## Slide 13
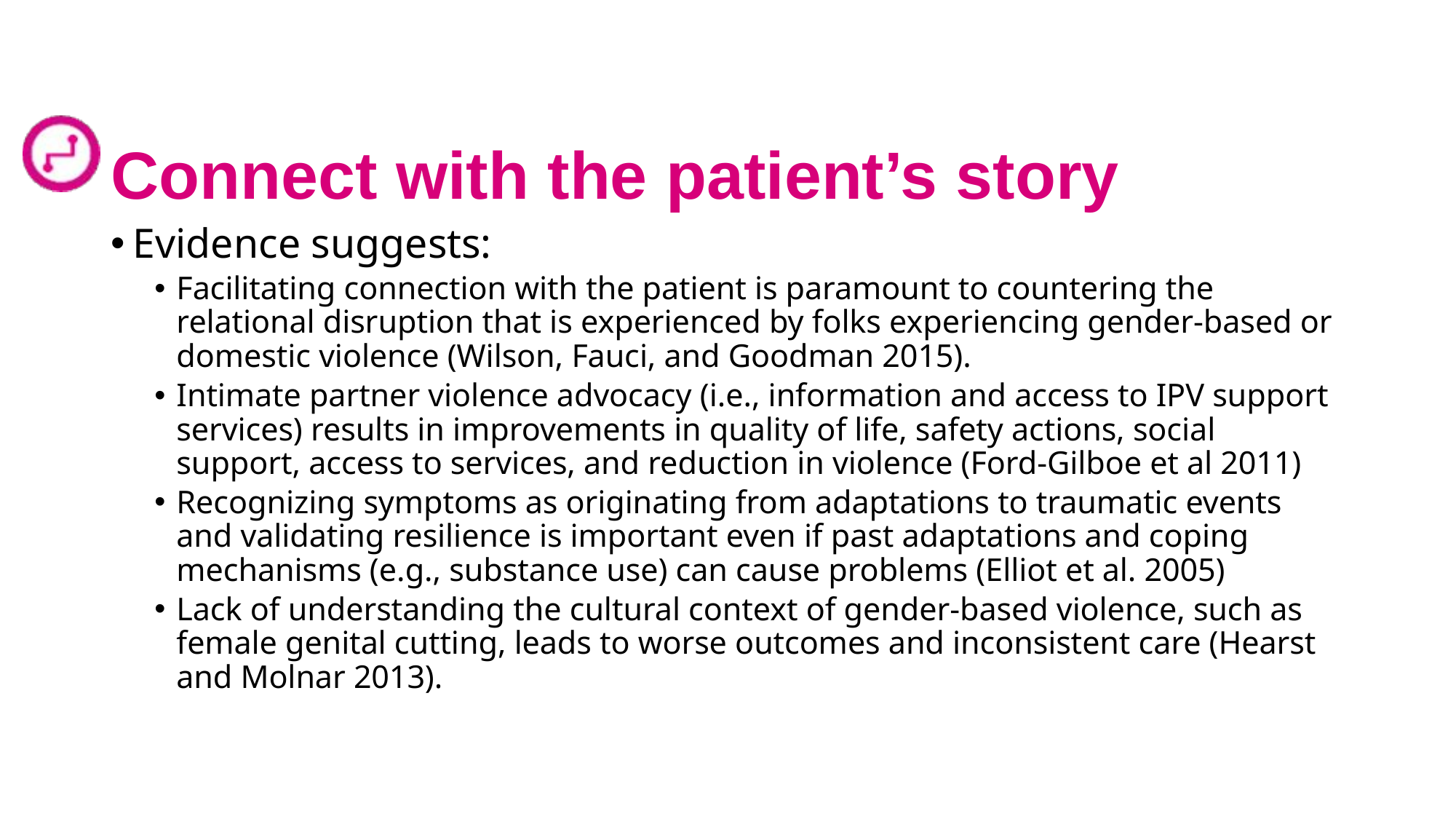

# Connect with the patient’s story
Evidence suggests:
Facilitating connection with the patient is paramount to countering the relational disruption that is experienced by folks experiencing gender-based or domestic violence (Wilson, Fauci, and Goodman 2015).
Intimate partner violence advocacy (i.e., information and access to IPV support services) results in improvements in quality of life, safety actions, social support, access to services, and reduction in violence (Ford-Gilboe et al 2011)
Recognizing symptoms as originating from adaptations to traumatic events and validating resilience is important even if past adaptations and coping mechanisms (e.g., substance use) can cause problems (Elliot et al. 2005)
Lack of understanding the cultural context of gender-based violence, such as female genital cutting, leads to worse outcomes and inconsistent care (Hearst and Molnar 2013).

## Slide 14
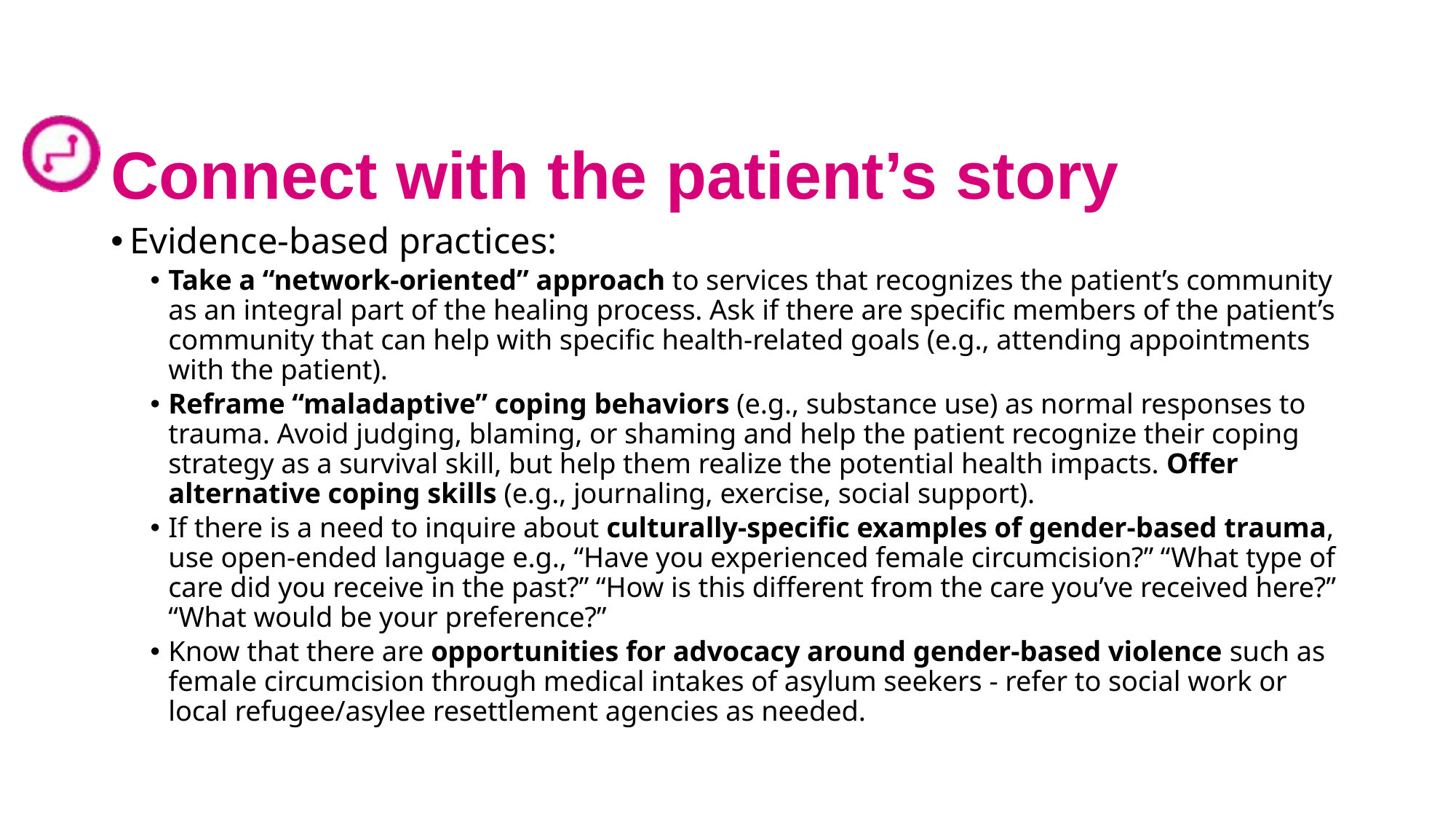

# Connect with the patient’s story
Evidence-based practices:
Take a “network-oriented” approach to services that recognizes the patient’s community as an integral part of the healing process. Ask if there are specific members of the patient’s community that can help with specific health-related goals (e.g., attending appointments with the patient).
Reframe “maladaptive” coping behaviors (e.g., substance use) as normal responses to trauma. Avoid judging, blaming, or shaming and help the patient recognize their coping strategy as a survival skill, but help them realize the potential health impacts. Offer alternative coping skills (e.g., journaling, exercise, social support).
If there is a need to inquire about culturally-specific examples of gender-based trauma, use open-ended language e.g., “Have you experienced female circumcision?” “What type of care did you receive in the past?” “How is this different from the care you’ve received here?” “What would be your preference?”
Know that there are opportunities for advocacy around gender-based violence such as female circumcision through medical intakes of asylum seekers - refer to social work or local refugee/asylee resettlement agencies as needed.

## Slide 15
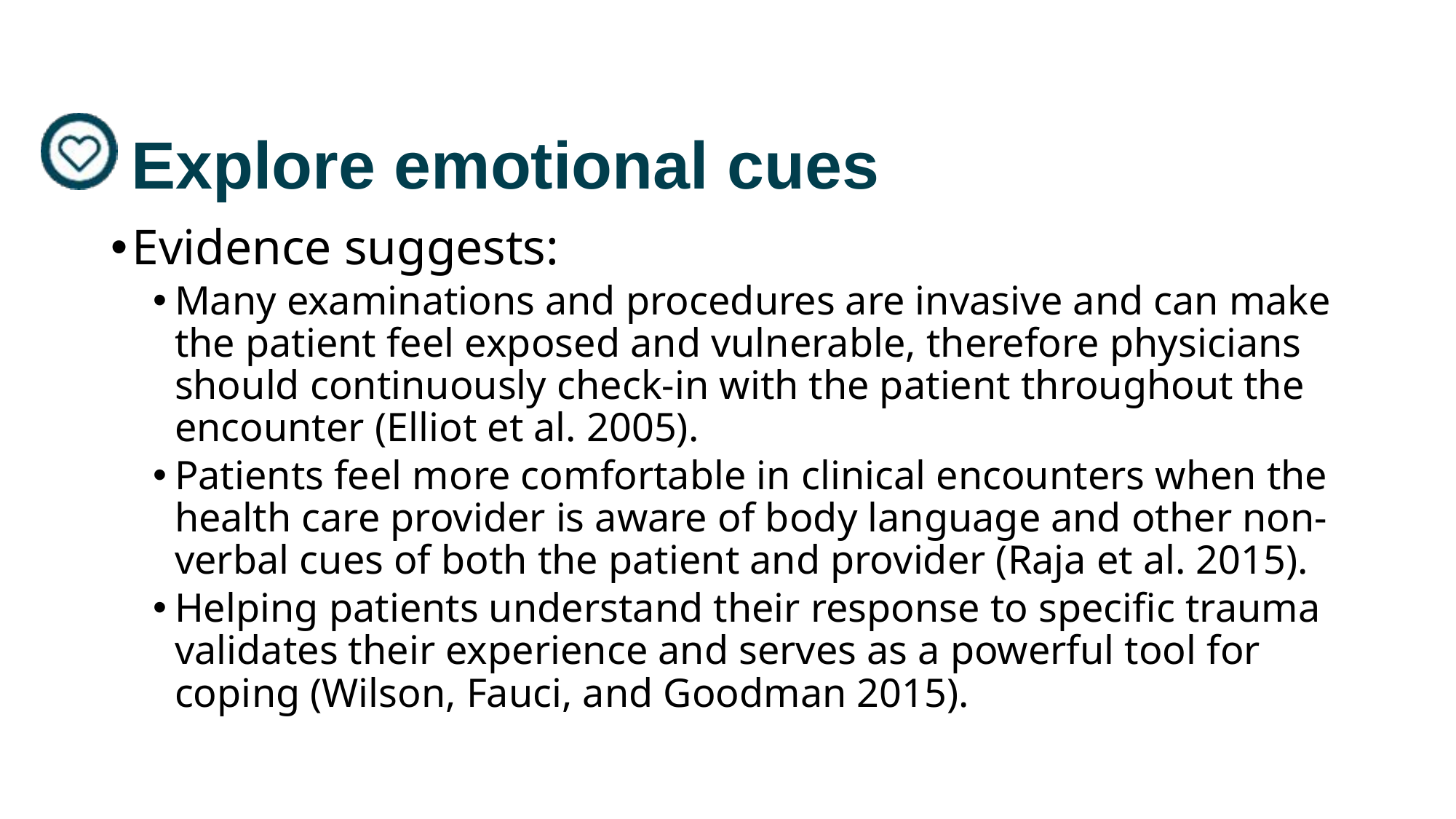

# Explore emotional cues
Evidence suggests:
Many examinations and procedures are invasive and can make the patient feel exposed and vulnerable, therefore physicians should continuously check-in with the patient throughout the encounter (Elliot et al. 2005).
Patients feel more comfortable in clinical encounters when the health care provider is aware of body language and other non-verbal cues of both the patient and provider (Raja et al. 2015).
Helping patients understand their response to specific trauma validates their experience and serves as a powerful tool for coping (Wilson, Fauci, and Goodman 2015).

## Slide 16
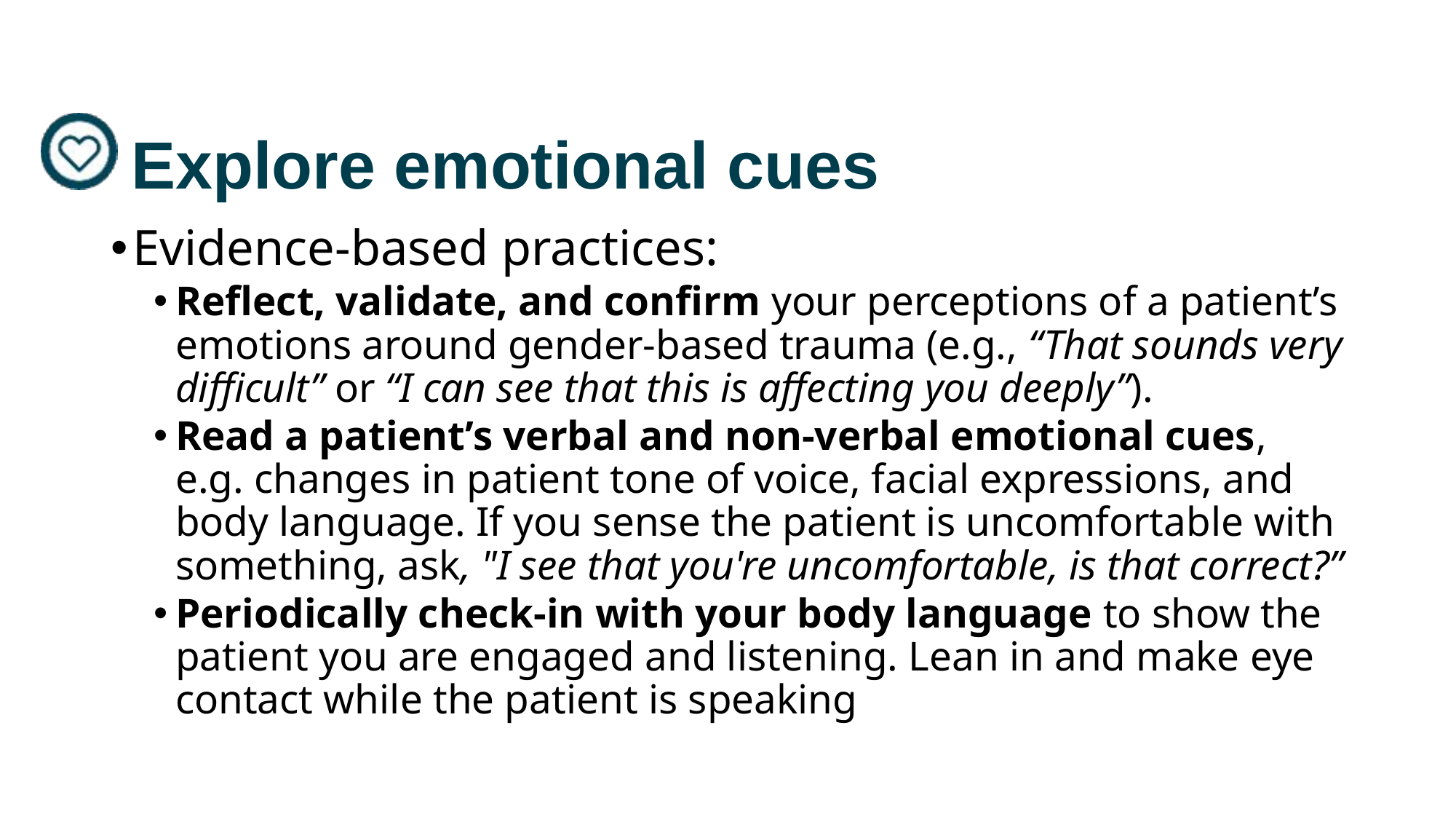

# Explore emotional cues
Evidence-based practices:
Reflect, validate, and confirm your perceptions of a patient’s emotions around gender-based trauma (e.g., “That sounds very difficult” or “I can see that this is affecting you deeply”).
Read a patient’s verbal and non-verbal emotional cues, e.g. changes in patient tone of voice, facial expressions, and body language. If you sense the patient is uncomfortable with something, ask, "I see that you're uncomfortable, is that correct?”
Periodically check-in with your body language to show the patient you are engaged and listening. Lean in and make eye contact while the patient is speaking

## Slide 17
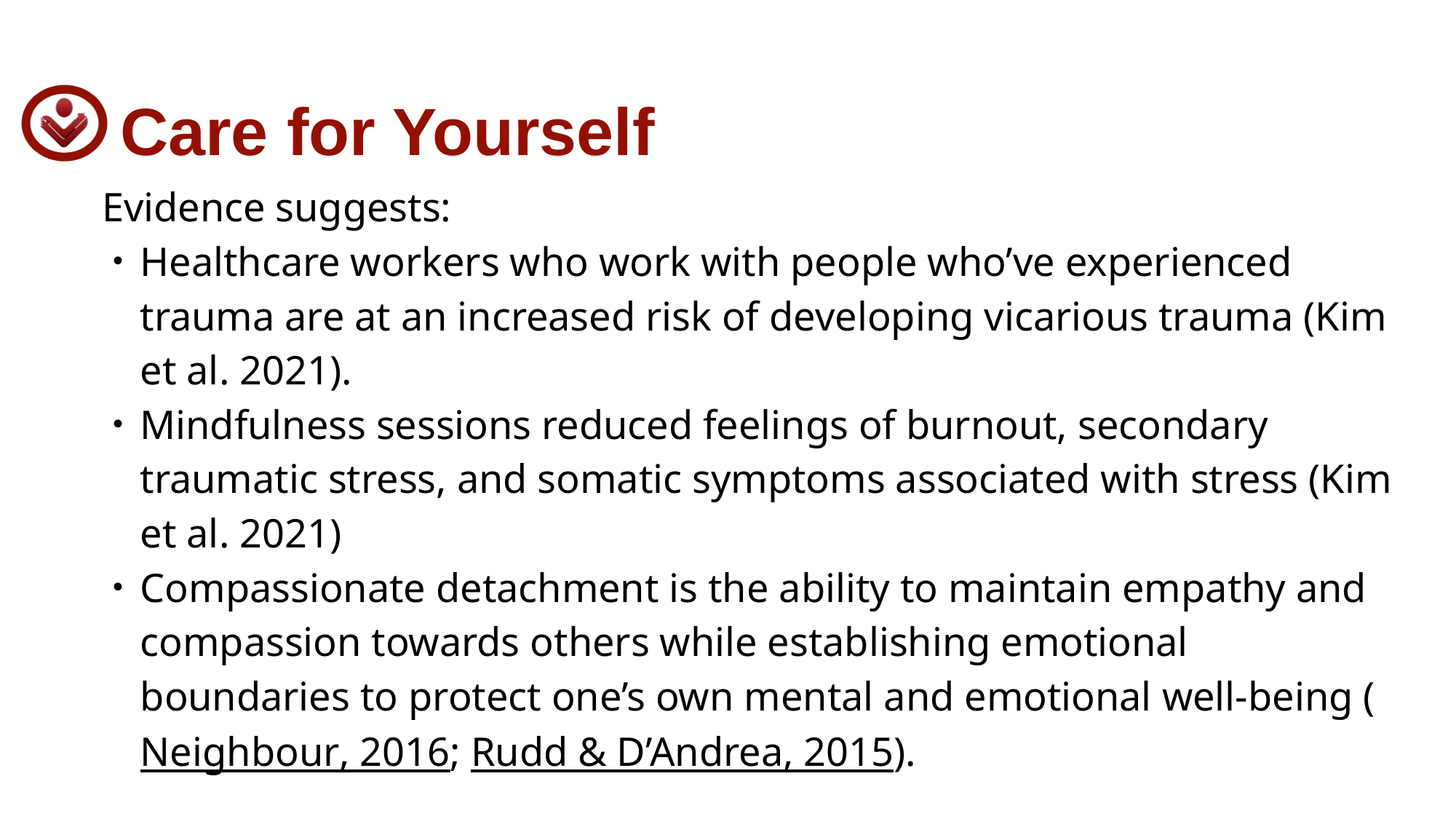

# Care for Yourself
Evidence suggests:
Healthcare workers who work with people who’ve experienced trauma are at an increased risk of developing vicarious trauma (Kim et al. 2021).
Mindfulness sessions reduced feelings of burnout, secondary traumatic stress, and somatic symptoms associated with stress (Kim et al. 2021)
Compassionate detachment is the ability to maintain empathy and compassion towards others while establishing emotional boundaries to protect one’s own mental and emotional well-being (Neighbour, 2016; Rudd & D’Andrea, 2015).

## Slide 18
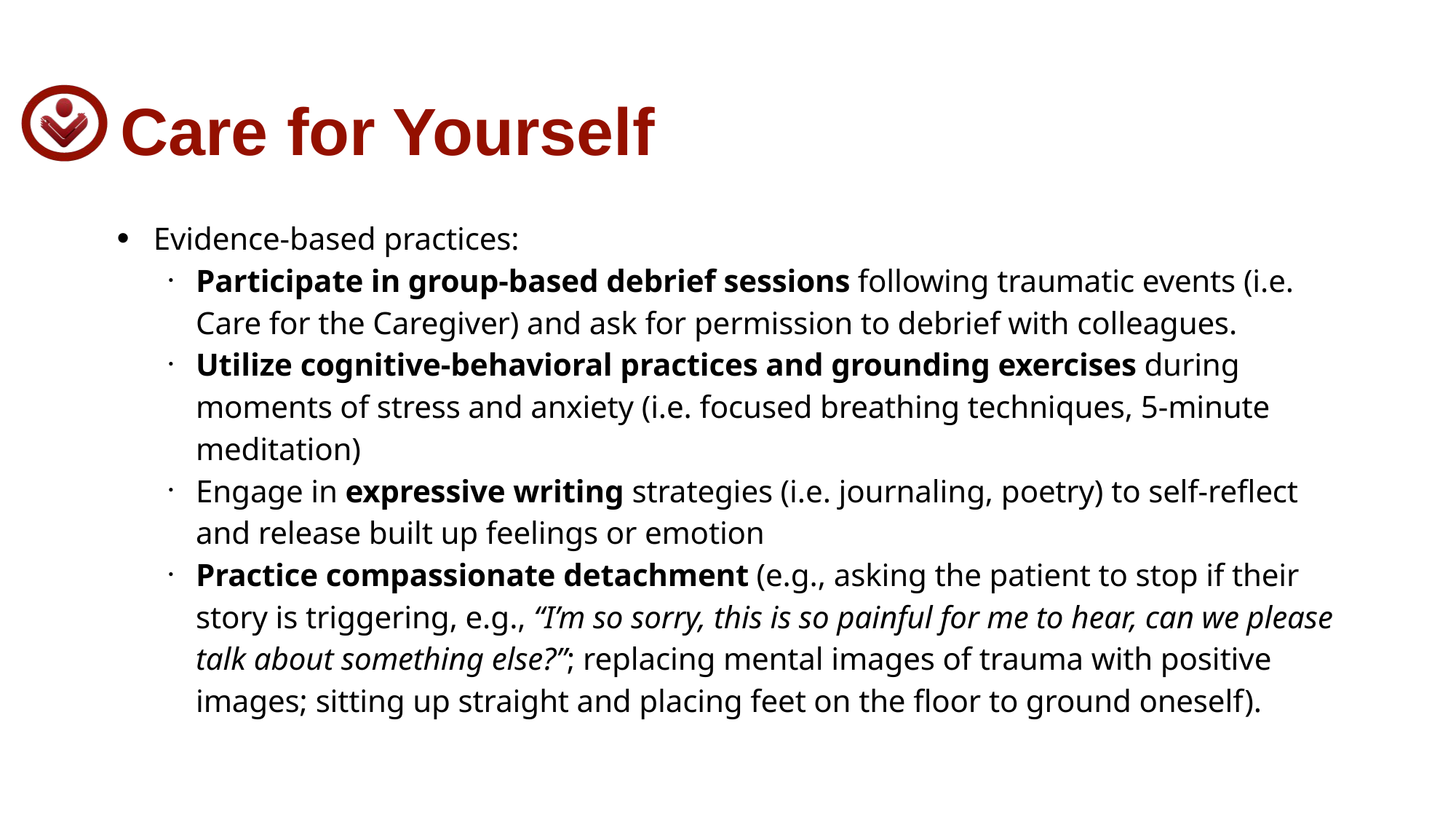

# Care for Yourself
Evidence-based practices:
Participate in group-based debrief sessions following traumatic events (i.e. Care for the Caregiver) and ask for permission to debrief with colleagues.
Utilize cognitive-behavioral practices and grounding exercises during moments of stress and anxiety (i.e. focused breathing techniques, 5-minute meditation)
Engage in expressive writing strategies (i.e. journaling, poetry) to self-reflect and release built up feelings or emotion
Practice compassionate detachment (e.g., asking the patient to stop if their story is triggering, e.g., “I’m so sorry, this is so painful for me to hear, can we please talk about something else?”; replacing mental images of trauma with positive images; sitting up straight and placing feet on the floor to ground oneself).

## Slide 19
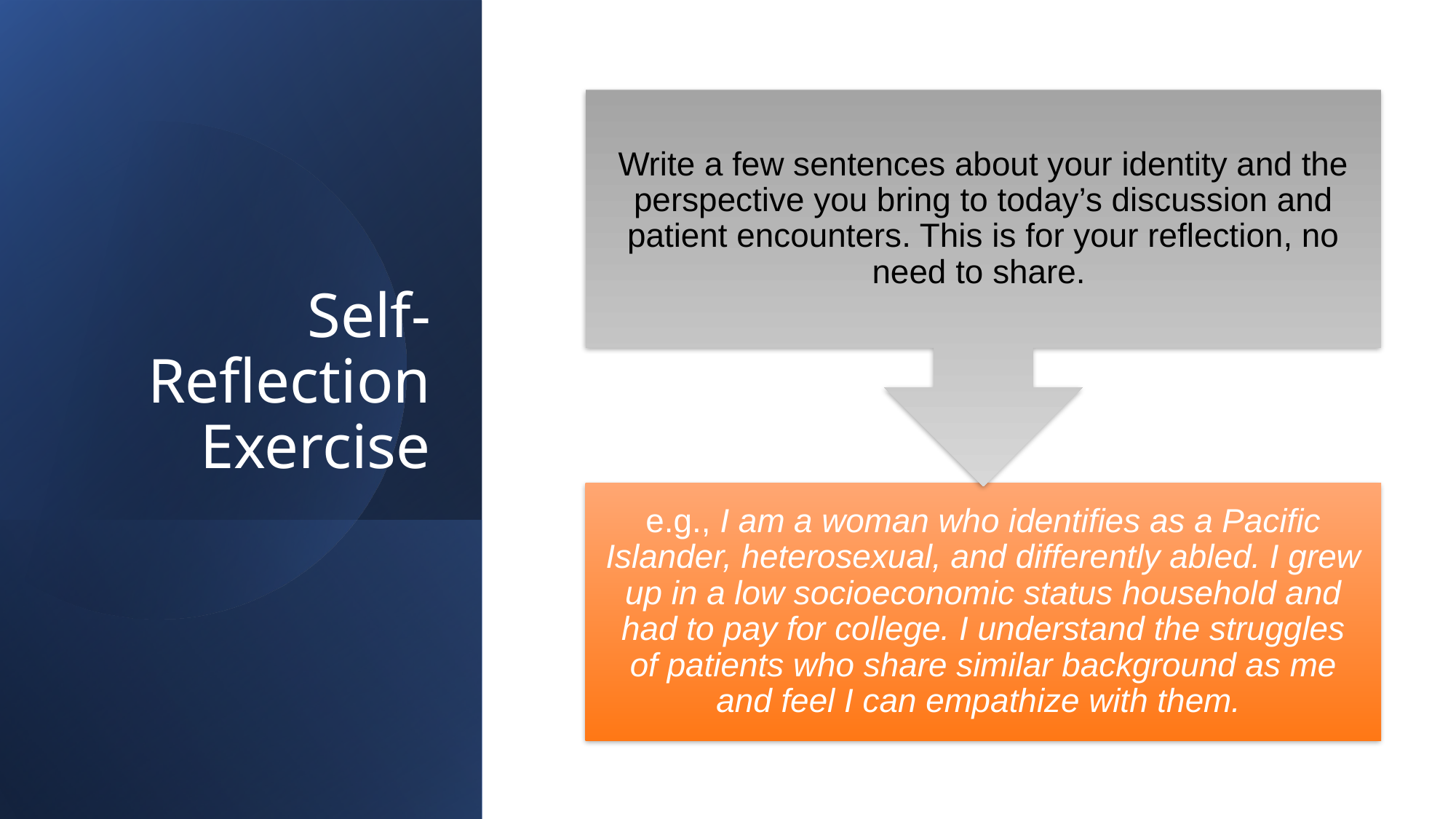

Write a few sentences about your identity and the perspective you bring to today’s discussion and patient encounters. This is for your reflection, no need to share.
e.g., I am a woman who identifies as a Pacific Islander, heterosexual, and differently abled. I grew up in a low socioeconomic status household and had to pay for college. I understand the struggles of patients who share similar background as me and feel I can empathize with them.
# Self-Reflection Exercise

## Slide 20
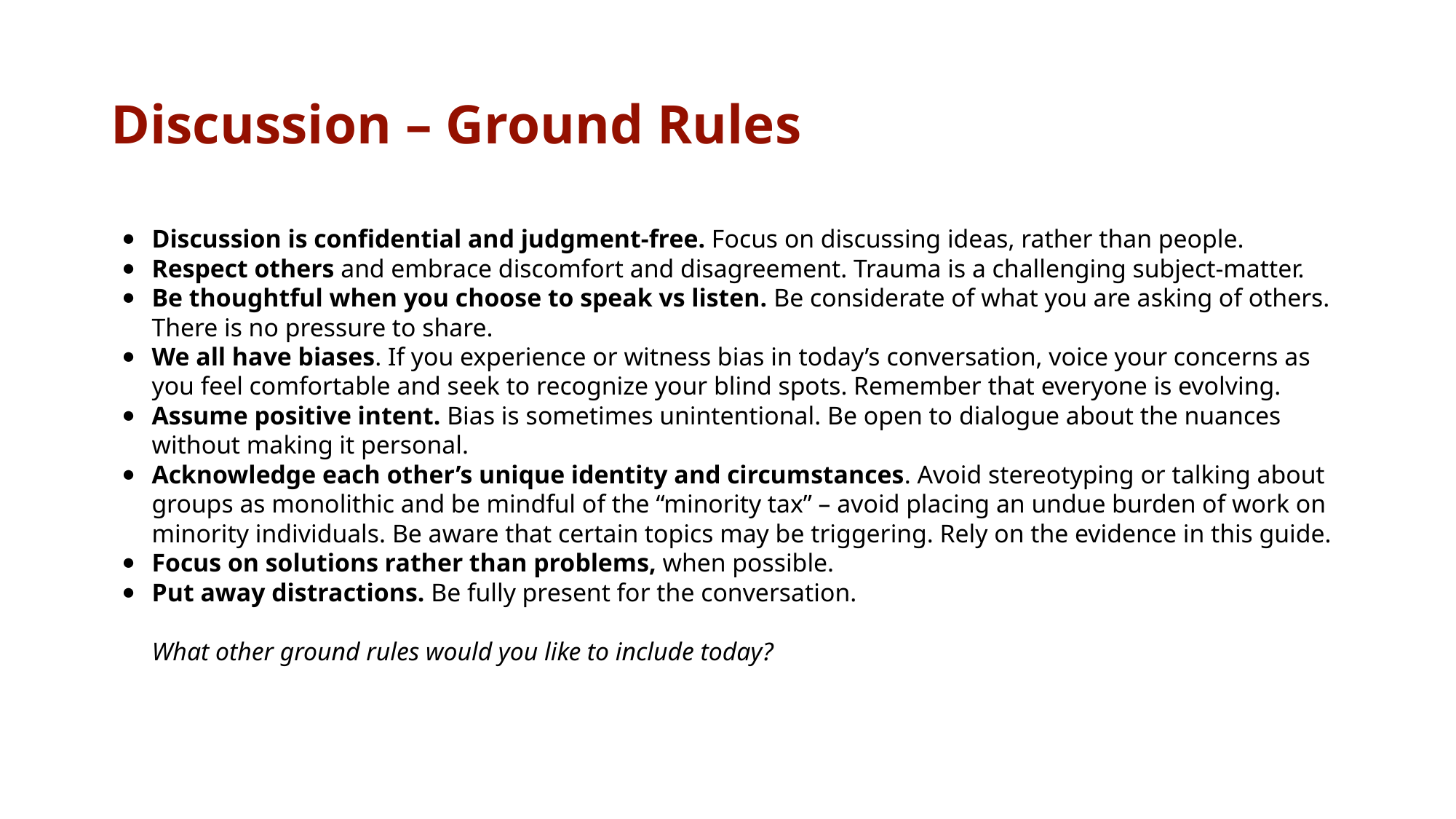

# Discussion – Ground Rules
Discussion is confidential and judgment-free. Focus on discussing ideas, rather than people.
Respect others and embrace discomfort and disagreement. Trauma is a challenging subject-matter.
Be thoughtful when you choose to speak vs listen. Be considerate of what you are asking of others. There is no pressure to share.
We all have biases. If you experience or witness bias in today’s conversation, voice your concerns as you feel comfortable and seek to recognize your blind spots. Remember that everyone is evolving.
Assume positive intent. Bias is sometimes unintentional. Be open to dialogue about the nuances without making it personal.
Acknowledge each other’s unique identity and circumstances. Avoid stereotyping or talking about groups as monolithic and be mindful of the “minority tax” – avoid placing an undue burden of work on minority individuals. Be aware that certain topics may be triggering. Rely on the evidence in this guide.
Focus on solutions rather than problems, when possible.
Put away distractions. Be fully present for the conversation.
What other ground rules would you like to include today?

## Slide 21
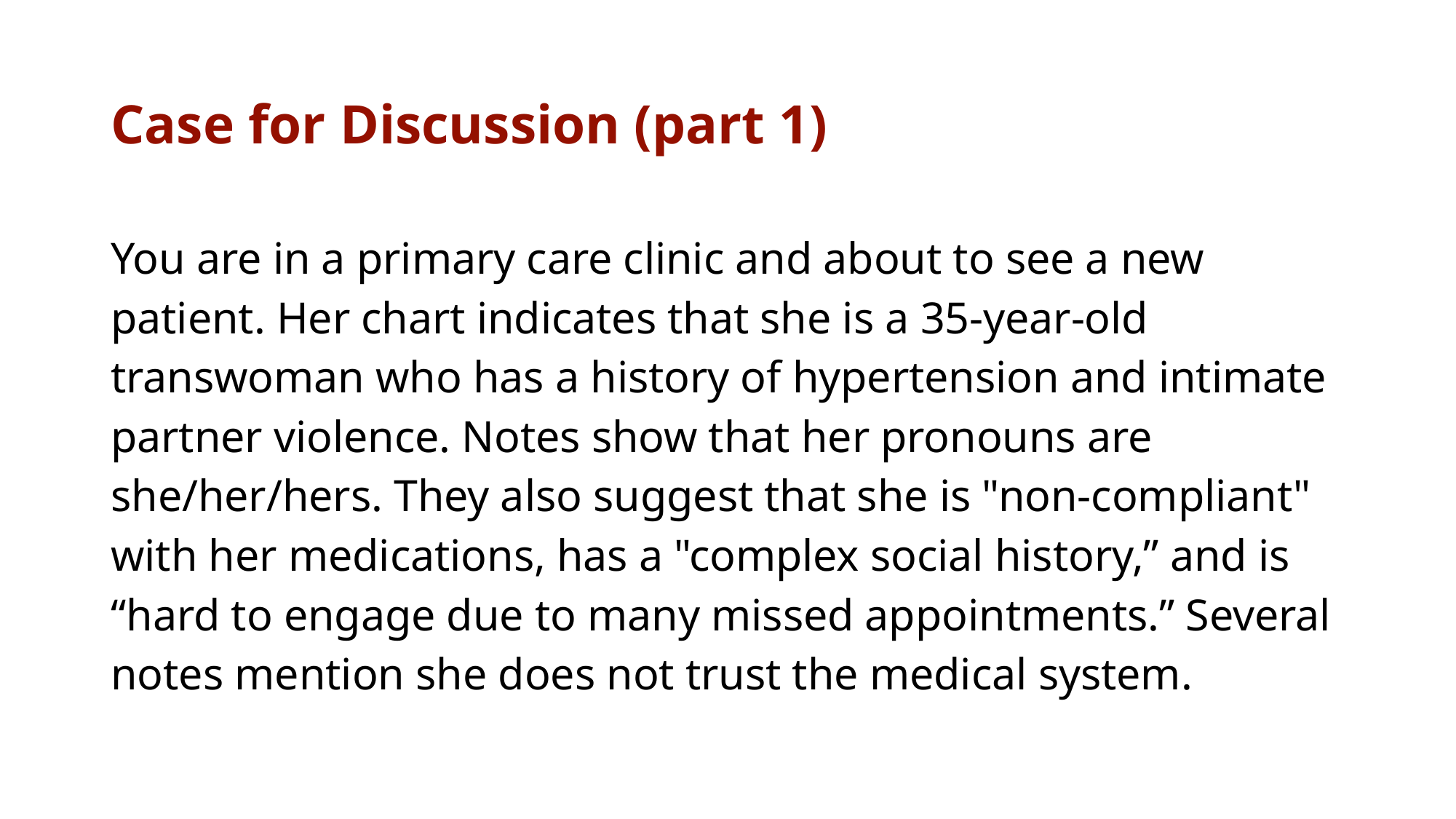

# Case for Discussion (part 1)
You are in a primary care clinic and about to see a new patient. Her chart indicates that she is a 35-year-old transwoman who has a history of hypertension and intimate partner violence. Notes show that her pronouns are she/her/hers. They also suggest that she is "non-compliant" with her medications, has a "complex social history,” and is “hard to engage due to many missed appointments.” Several notes mention she does not trust the medical system.

## Slide 22
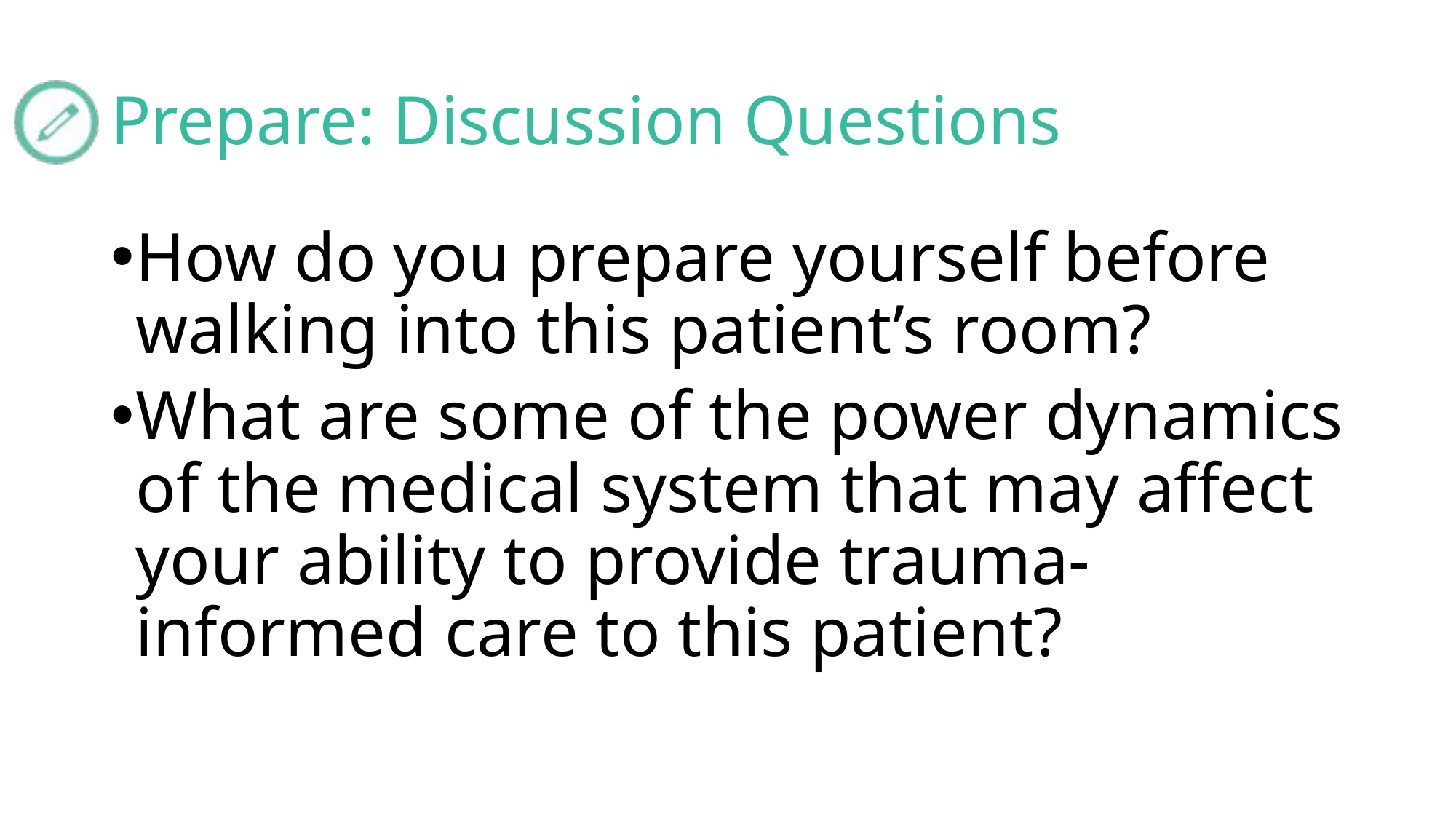

# Prepare: Discussion Questions
How do you prepare yourself before walking into this patient’s room?
What are some of the power dynamics of the medical system that may affect your ability to provide trauma-informed care to this patient?

## Slide 23
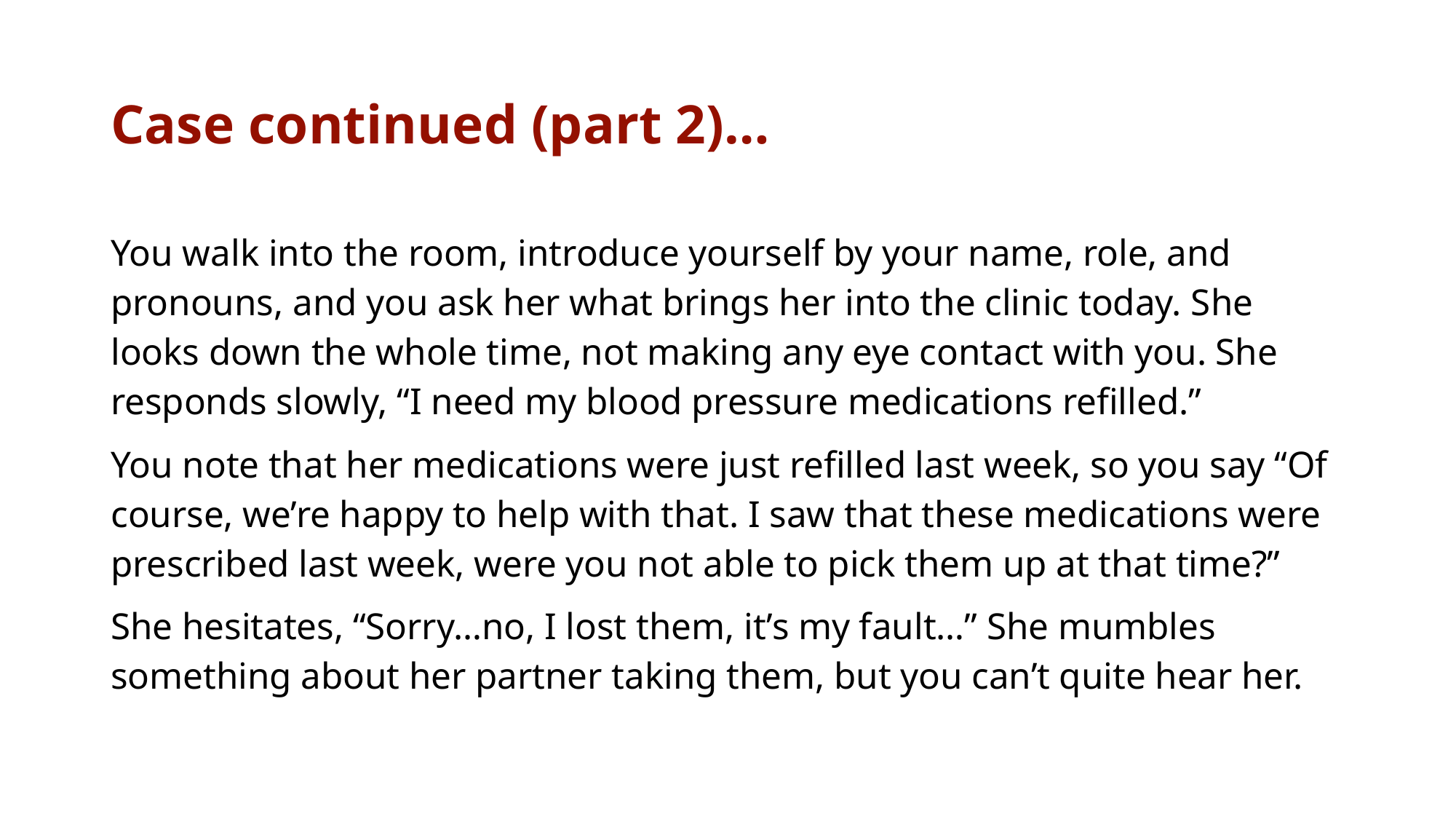

# Case continued (part 2)…
You walk into the room, introduce yourself by your name, role, and pronouns, and you ask her what brings her into the clinic today. She looks down the whole time, not making any eye contact with you. She responds slowly, “I need my blood pressure medications refilled.”
You note that her medications were just refilled last week, so you say “Of course, we’re happy to help with that. I saw that these medications were prescribed last week, were you not able to pick them up at that time?”
She hesitates, “Sorry…no, I lost them, it’s my fault…” She mumbles something about her partner taking them, but you can’t quite hear her.

## Slide 24
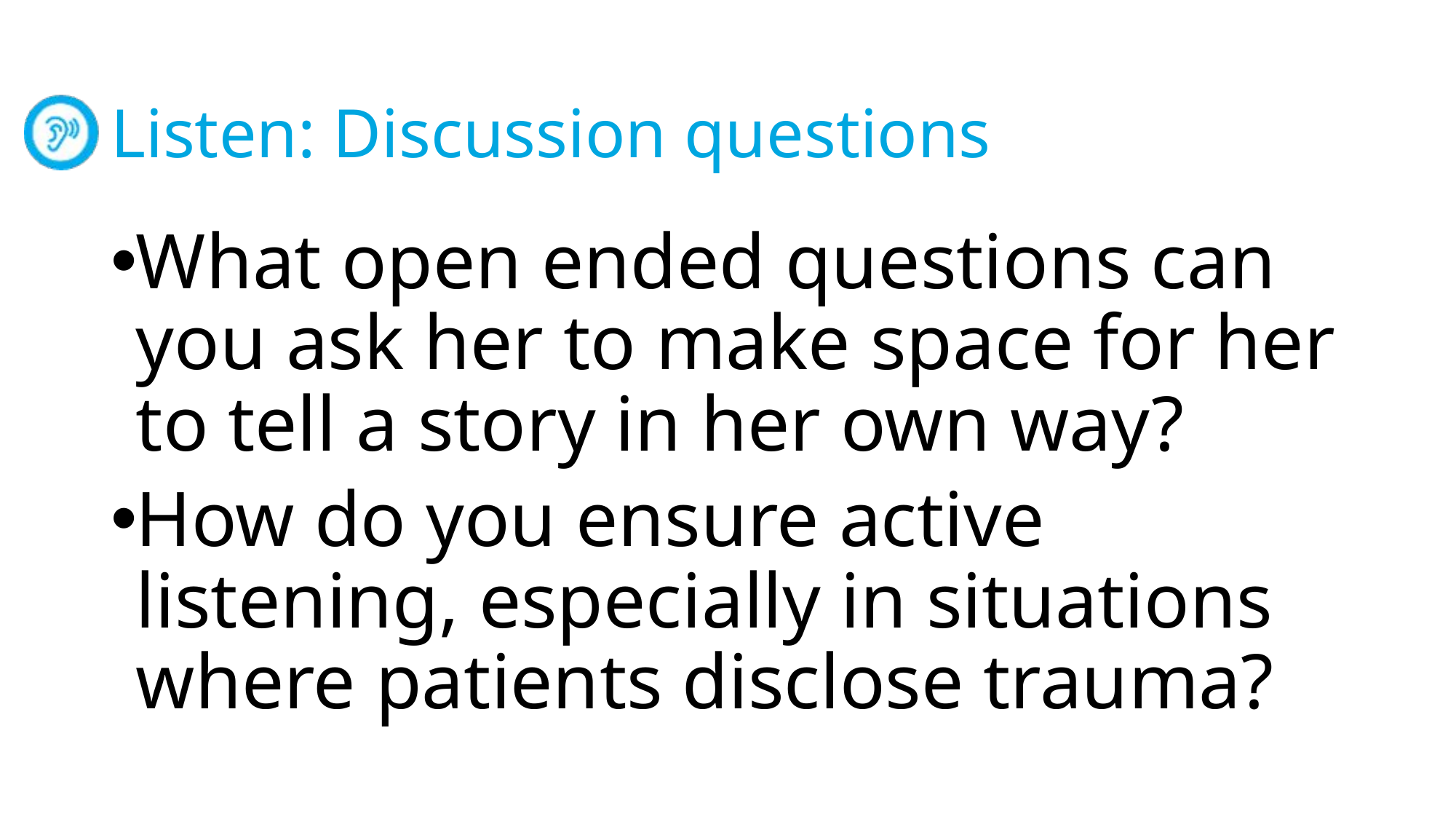

# Listen: Discussion questions
What open ended questions can you ask her to make space for her to tell a story in her own way?
How do you ensure active listening, especially in situations where patients disclose trauma?

## Slide 25
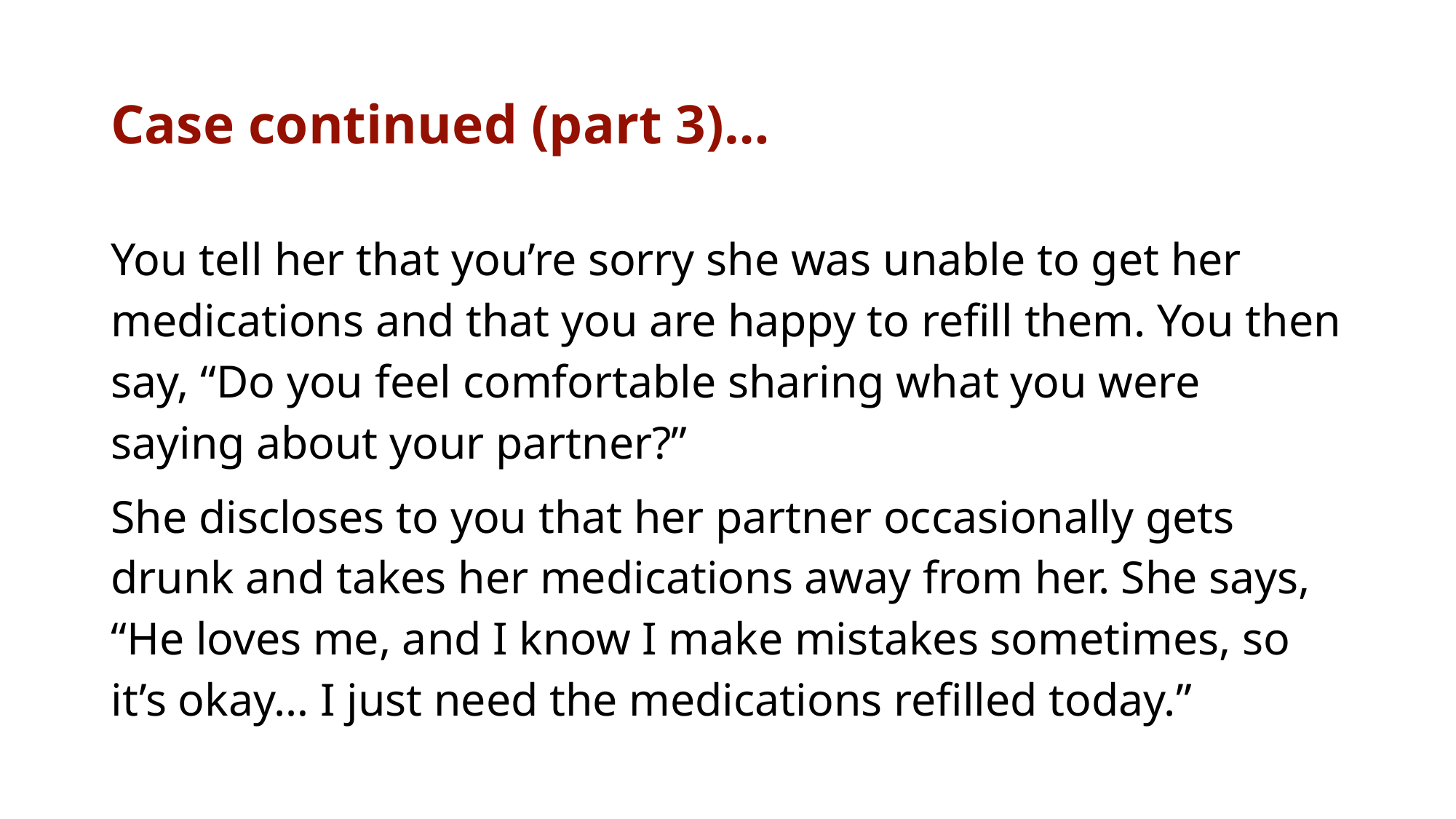

# Case continued (part 3)…
You tell her that you’re sorry she was unable to get her medications and that you are happy to refill them. You then say, “Do you feel comfortable sharing what you were saying about your partner?”
She discloses to you that her partner occasionally gets drunk and takes her medications away from her. She says, “He loves me, and I know I make mistakes sometimes, so it’s okay… I just need the medications refilled today.”

## Slide 26
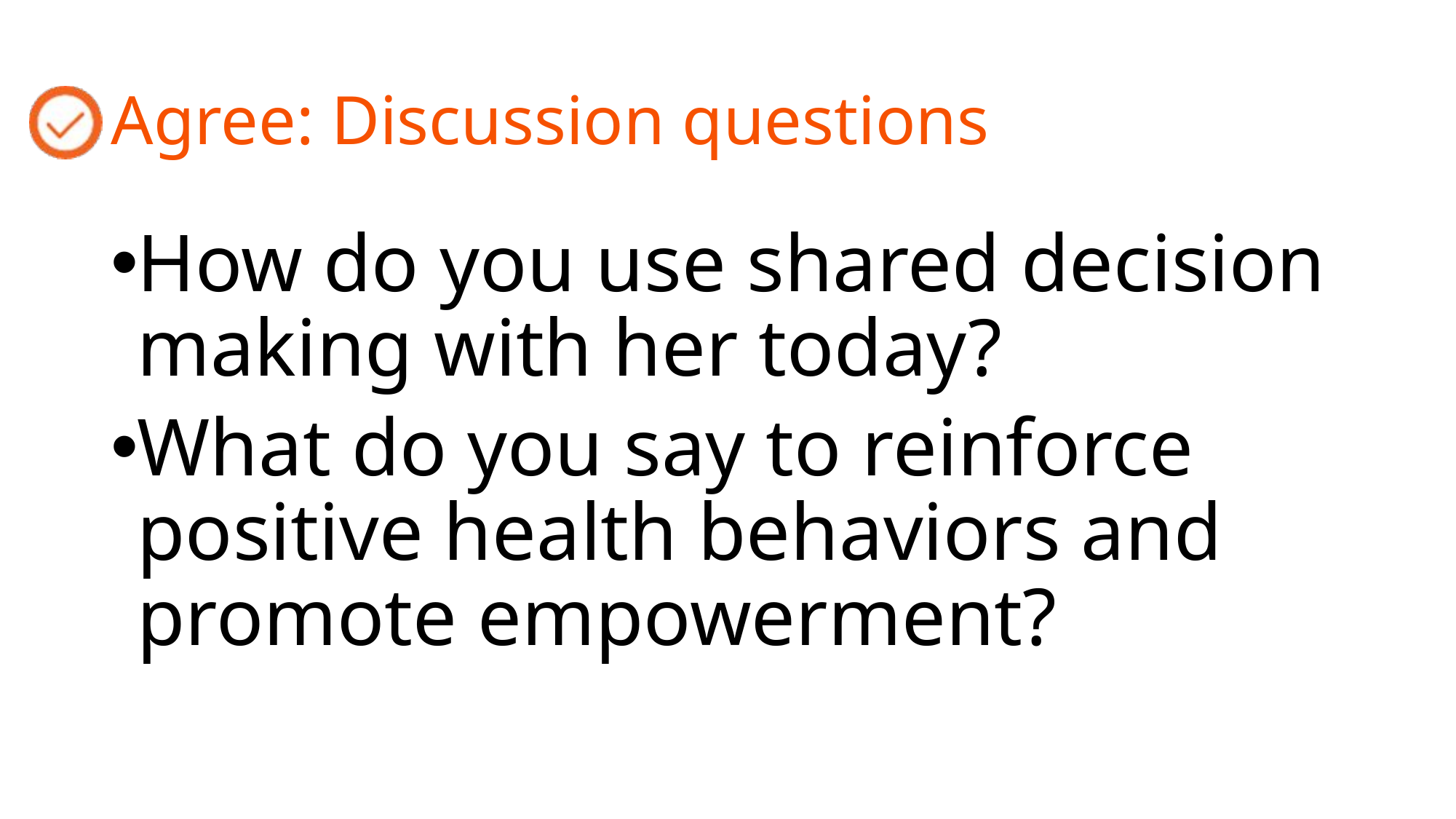

# Agree: Discussion questions
How do you use shared decision making with her today?
What do you say to reinforce positive health behaviors and promote empowerment?

## Slide 27
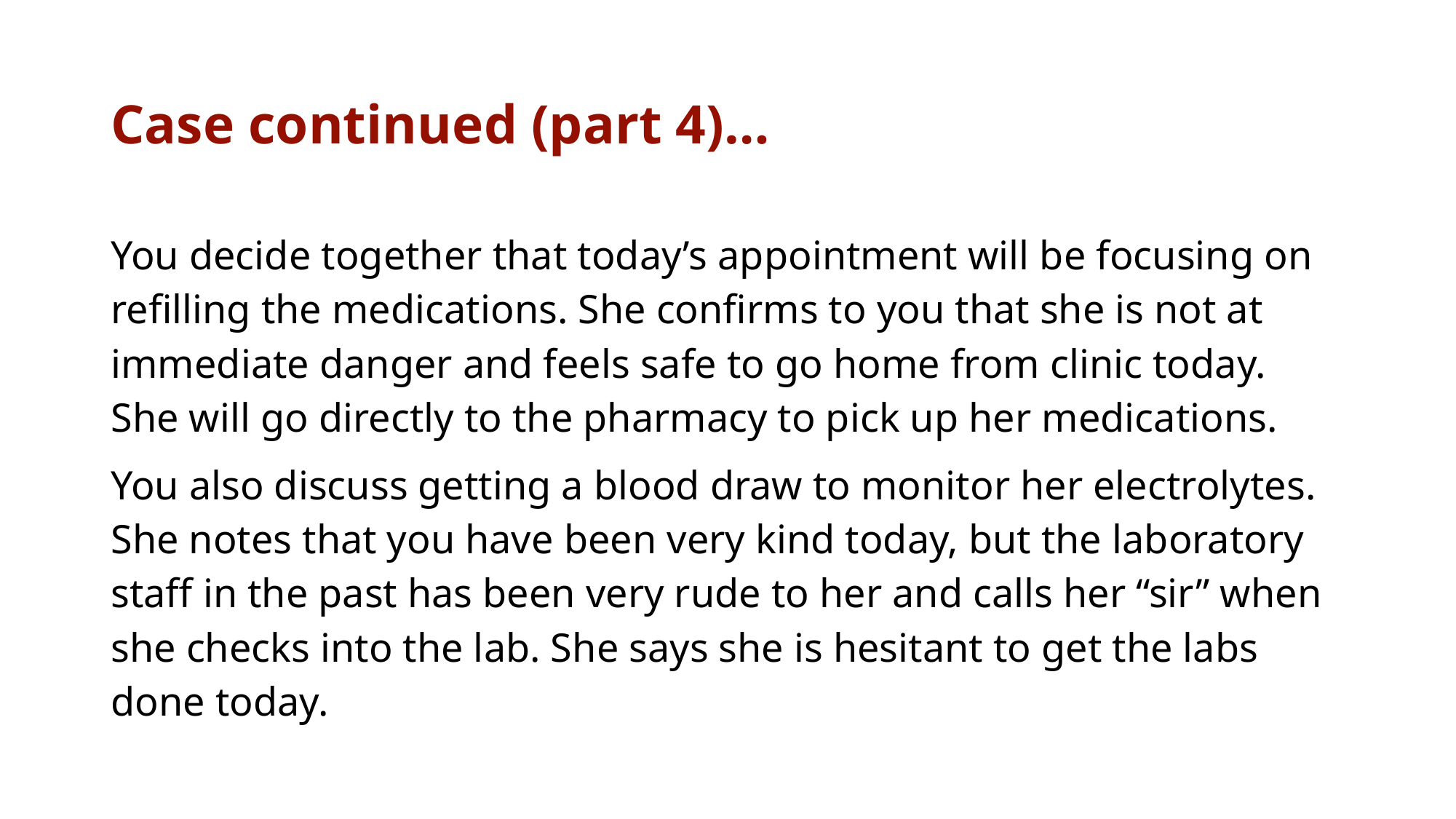

# Case continued (part 4)…
You decide together that today’s appointment will be focusing on refilling the medications. She confirms to you that she is not at immediate danger and feels safe to go home from clinic today. She will go directly to the pharmacy to pick up her medications.
You also discuss getting a blood draw to monitor her electrolytes. She notes that you have been very kind today, but the laboratory staff in the past has been very rude to her and calls her “sir” when she checks into the lab. She says she is hesitant to get the labs done today.

## Slide 28
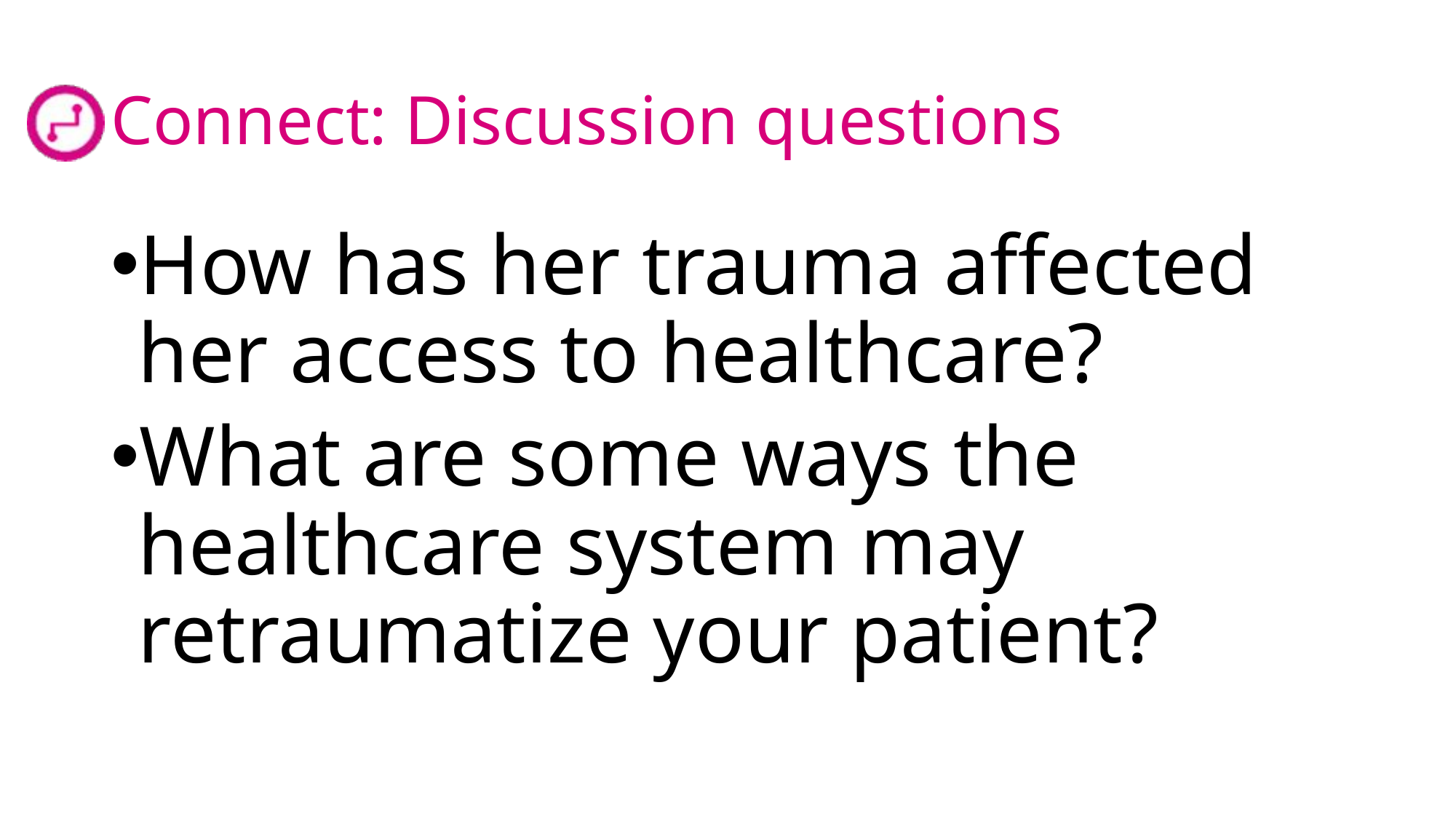

# Connect: Discussion questions
How has her trauma affected her access to healthcare?
What are some ways the healthcare system may retraumatize your patient?

## Slide 29
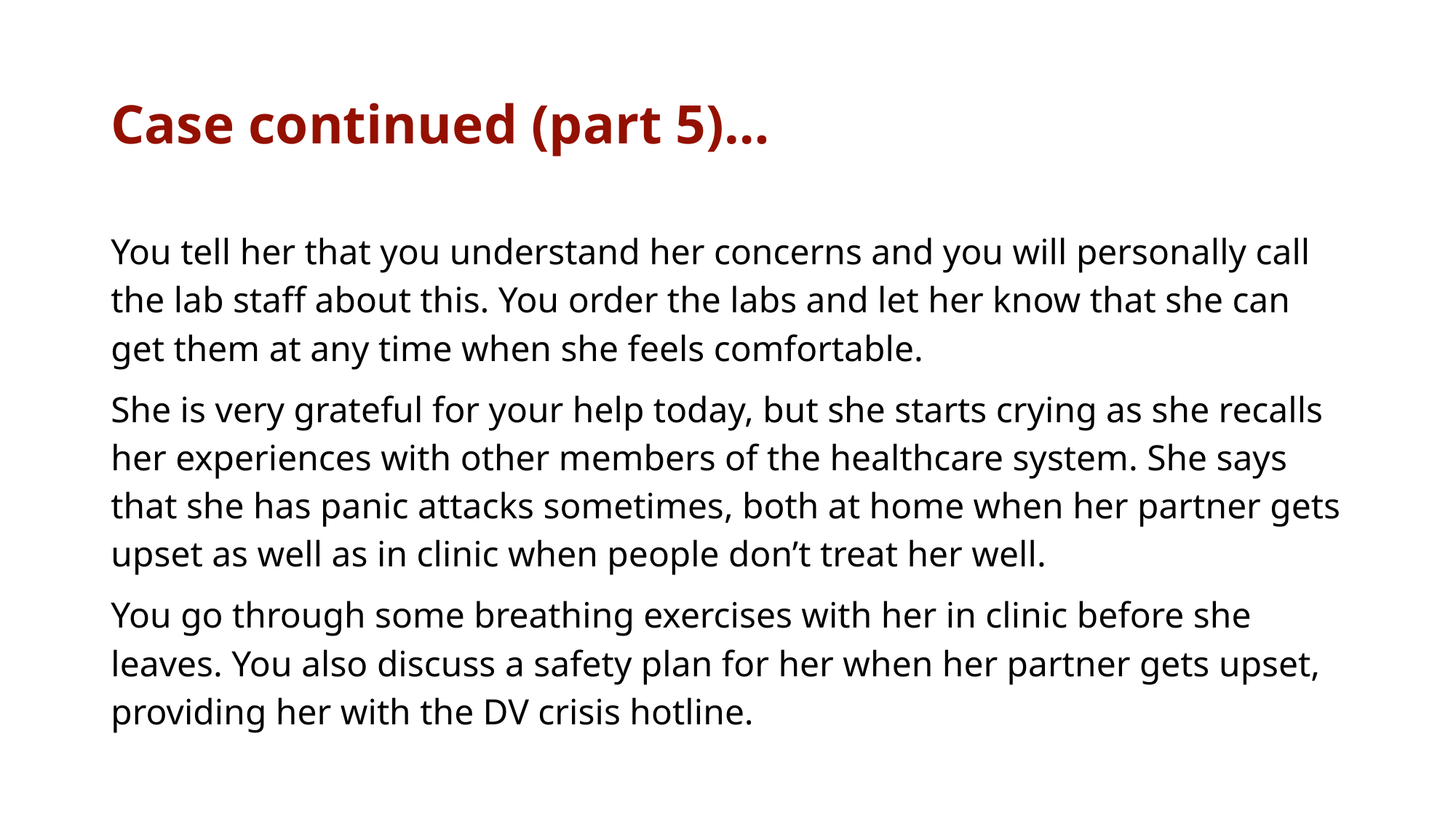

# Case continued (part 5)…
You tell her that you understand her concerns and you will personally call the lab staff about this. You order the labs and let her know that she can get them at any time when she feels comfortable.
She is very grateful for your help today, but she starts crying as she recalls her experiences with other members of the healthcare system. She says that she has panic attacks sometimes, both at home when her partner gets upset as well as in clinic when people don’t treat her well.
You go through some breathing exercises with her in clinic before she leaves. You also discuss a safety plan for her when her partner gets upset, providing her with the DV crisis hotline.

## Slide 30
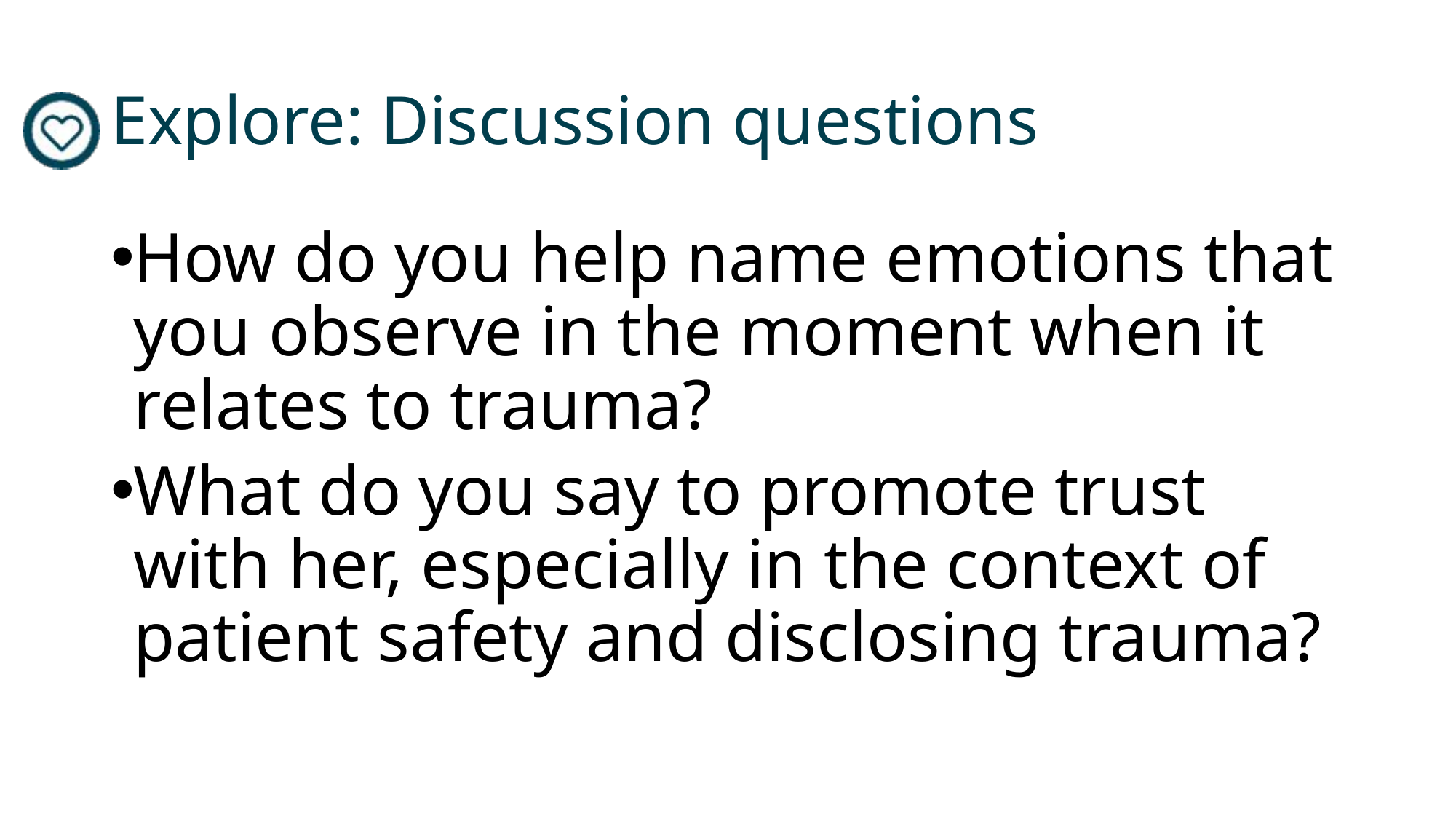

# Explore: Discussion questions
How do you help name emotions that you observe in the moment when it relates to trauma?
What do you say to promote trust with her, especially in the context of patient safety and disclosing trauma?

## Slide 31
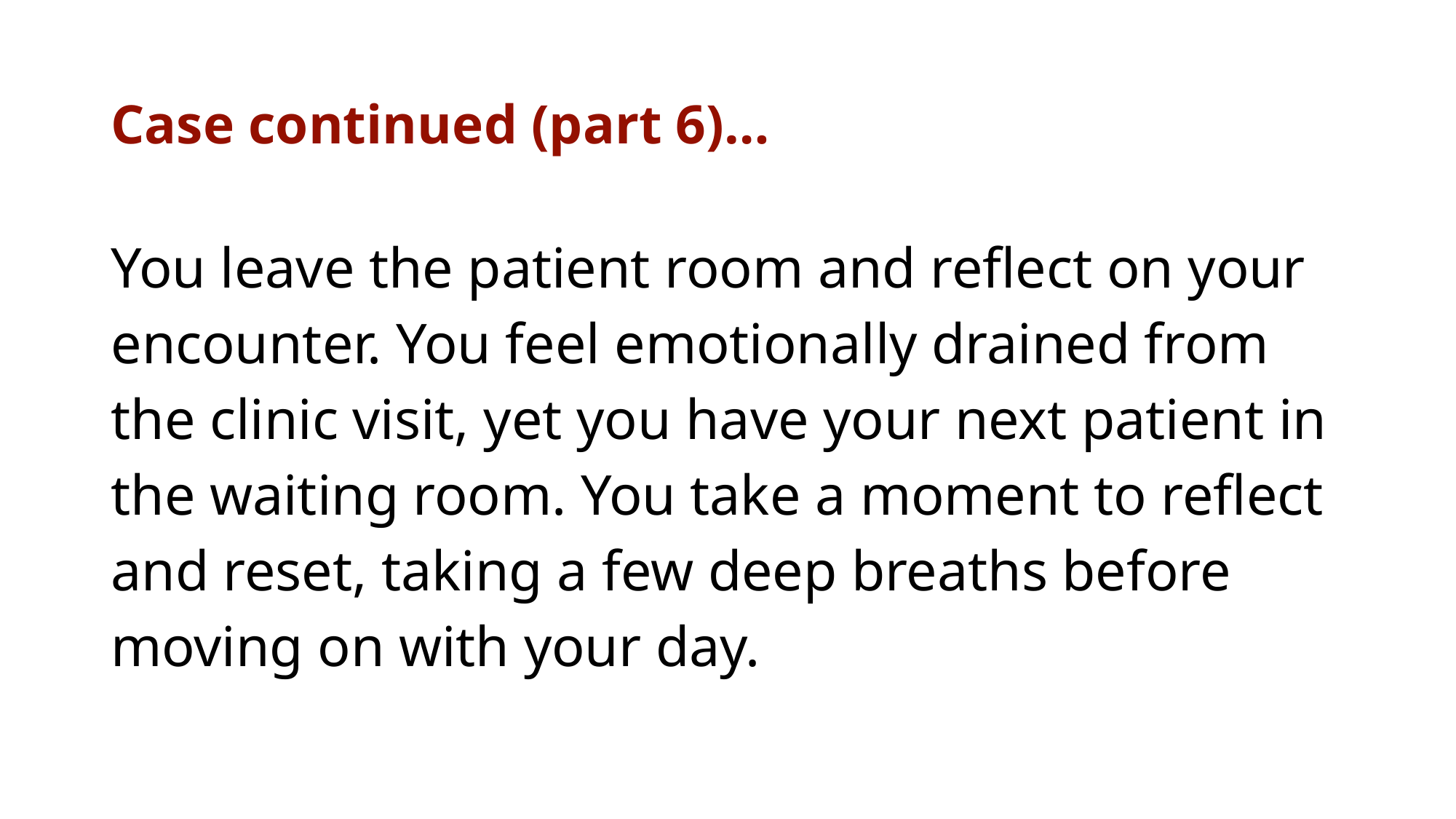

# Case continued (part 6)…
You leave the patient room and reflect on your encounter. You feel emotionally drained from the clinic visit, yet you have your next patient in the waiting room. You take a moment to reflect and reset, taking a few deep breaths before moving on with your day.

## Slide 32
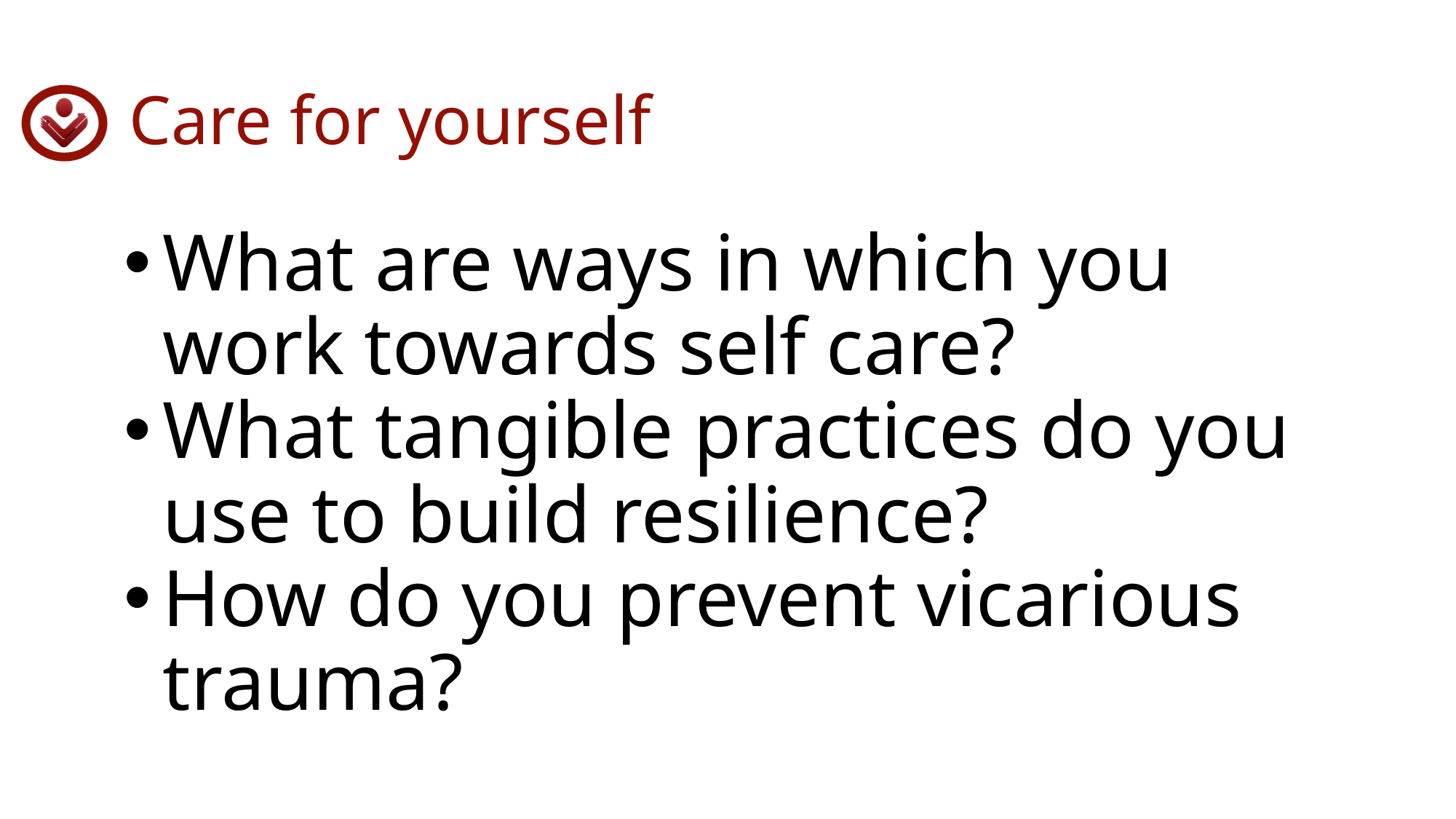

# Care for yourself
What are ways in which you work towards self care?
What tangible practices do you use to build resilience?
How do you prevent vicarious trauma?

## Slide 33
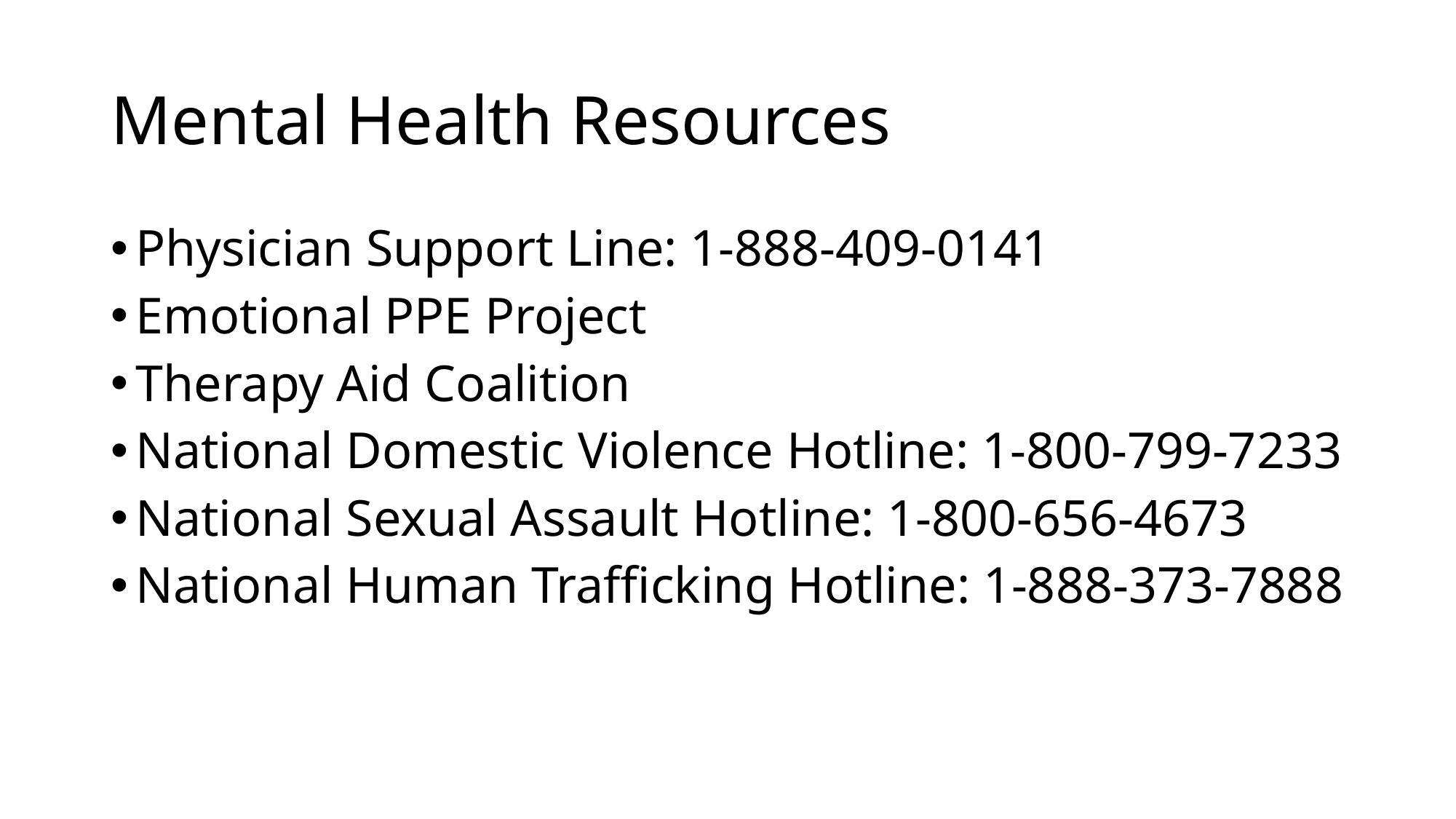

# Mental Health Resources
Physician Support Line: 1-888-409-0141
Emotional PPE Project
Therapy Aid Coalition
National Domestic Violence Hotline: 1-800-799-7233
National Sexual Assault Hotline: 1-800-656-4673
National Human Trafficking Hotline: 1-888-373-7888

## Slide 34
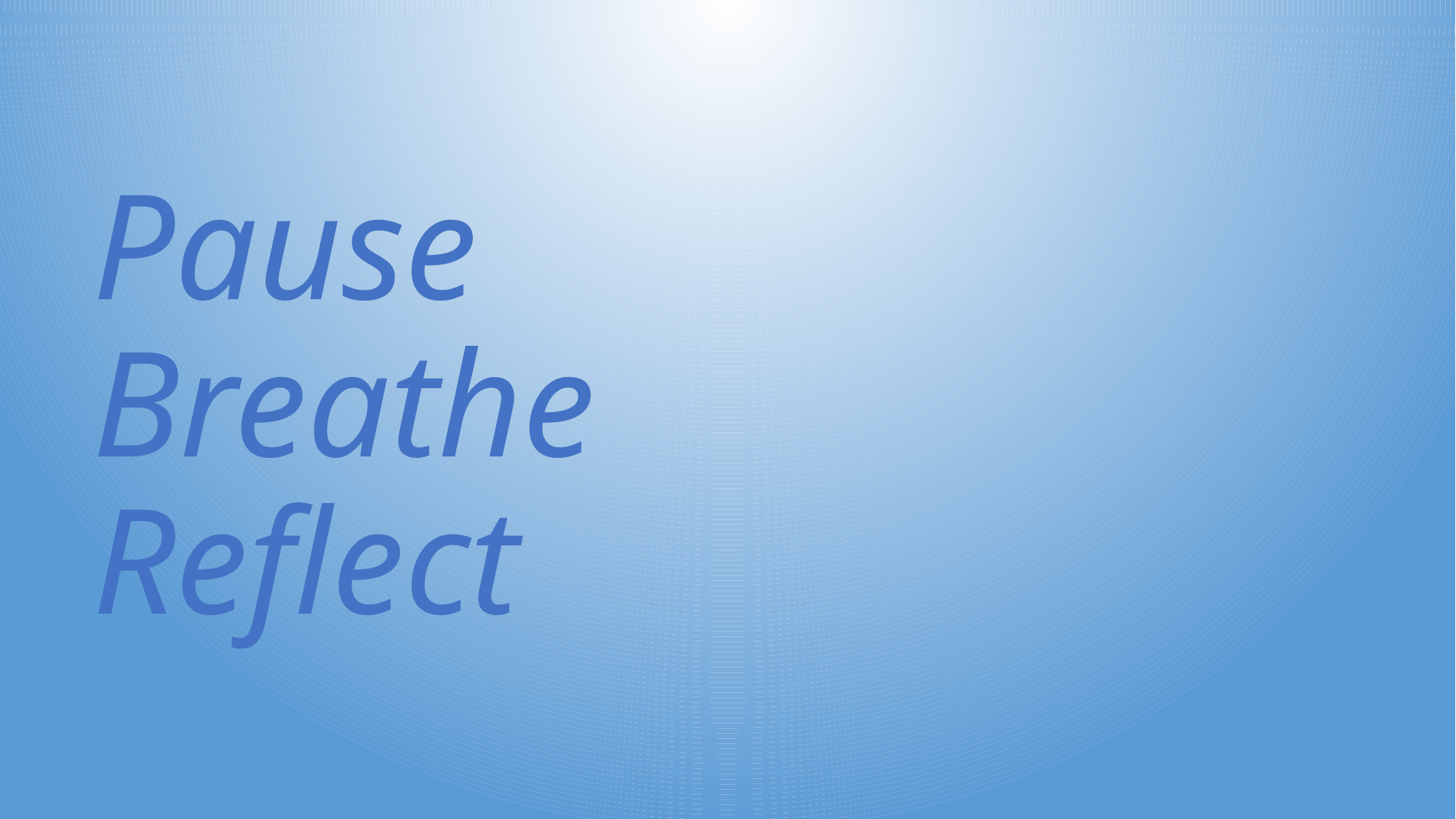

# Pause Breathe Reflect

## Slide 35
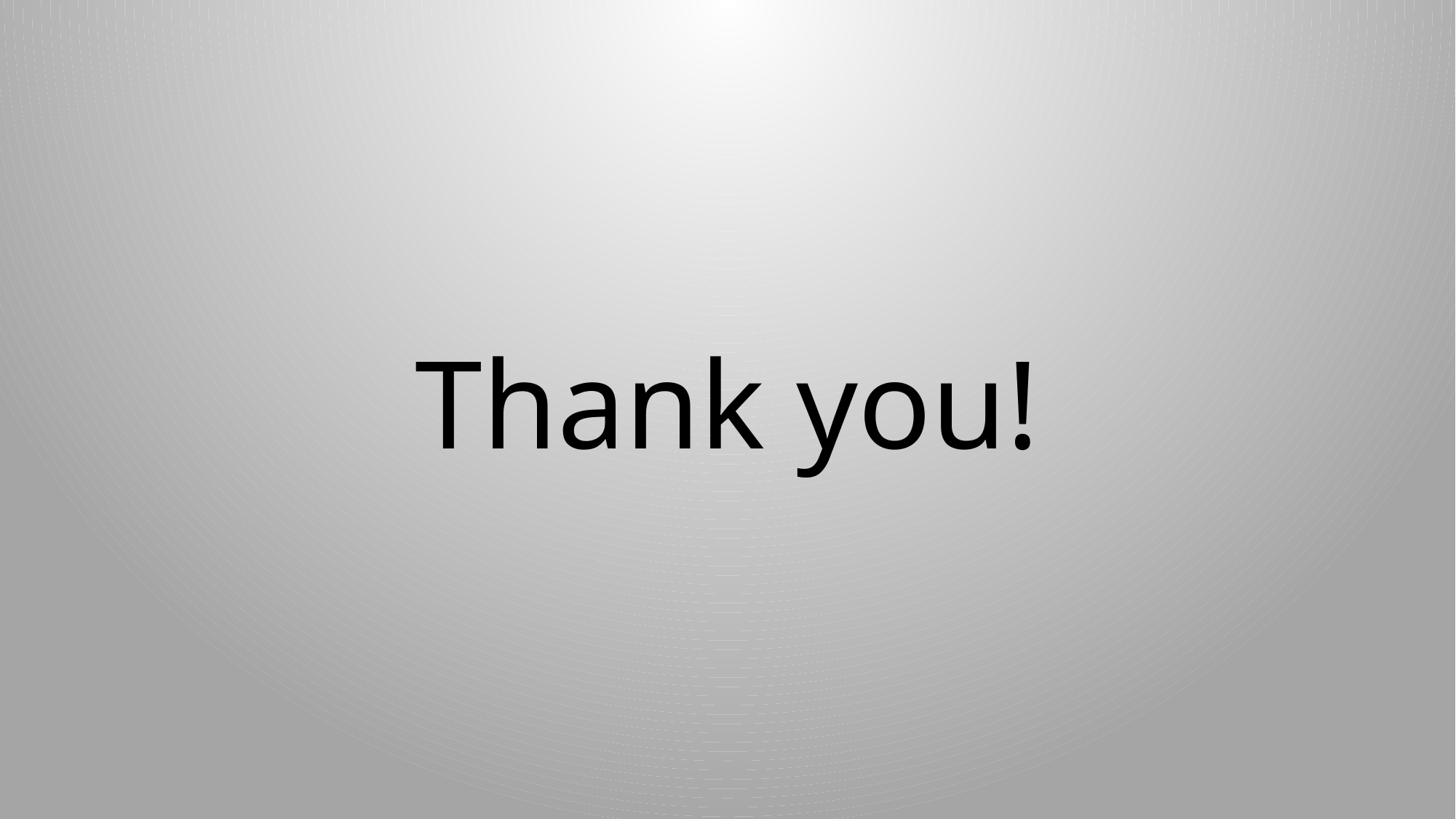

# Thank you!
